# Does a presentation’s medium affect its message? PowerPoint, Prezi, and oral presentations

**DOI:** 10.1371/journal.pone.0178774

**Published:** 2017-07-05

**Authors:** Samuel T. Moulton, Selen Türkay, Stephen M. Kosslyn

**Affiliations:** 1 Department of Psychology, Harvard University, Cambridge, Massachusetts, United States of America; 2 Harvard Initiative for Learning and Teaching, Harvard University, Cambridge, Massachusetts, United States of America; 3 Minerva Schools at the Keck Graduate Institute, San Francisco, California, United States of America; University of Akron, UNITED STATES

## Abstract

Despite the prevalence of PowerPoint in professional and educational presentations, surprisingly little is known about how effective such presentations are. All else being equal, are PowerPoint presentations better than purely oral presentations or those that use alternative software tools? To address this question we recreated a real-world business scenario in which individuals presented to a corporate board. Participants (playing the role of the presenter) were randomly assigned to create PowerPoint, Prezi, or oral presentations, and then actually delivered the presentation live to other participants (playing the role of corporate executives). Across two experiments and on a variety of dimensions, participants evaluated PowerPoint presentations comparably to oral presentations, but evaluated Prezi presentations more favorably than both PowerPoint and oral presentations. There was some evidence that participants who viewed different types of presentations came to different conclusions about the business scenario, but no evidence that they remembered or comprehended the scenario differently. We conclude that the observed effects of presentation format are not merely the result of novelty, bias, experimenter-, or software-specific characteristics, but instead reveal a communication preference for using the panning-and-zooming animations that characterize Prezi presentations.

## Introduction

How do the characteristics of a communication medium affect its messages? This question has been the subject of much philosophical and empirical inquiry, with some (e.g., [1]) claiming that the medium determines the message (“the medium is the message”), others (e.g., [2]) claiming that characteristics of a medium affect the message, and others claiming that the medium and message are separable (e.g.,[3,4]). As psychologists, we ask: What mental mechanisms underlie effective communication and how can presenters leverage these mechanisms to communicate better? These questions—at the intersection of psychology and communication practice—motivate this research.

That said, the relative efficacy of different communication media or technologies informs the primary questions of interest. If we can demonstrate that oral presentations are less or more effective than those that rely on presentation software—or that presenters who use one type of presentation software tend to be more effective than those who use another—then we advance our psychological and practical understanding of effective communication. Thus, in the tradition of use-inspired basic research [5]—and as a means to an end, rather than an end unto itself—we compare the effectiveness of three commonly-used formats for communication: oral, PowerPoint, and Prezi presentations.

We focused on presentations because they populate our academic, professional, and even personal lives in the form of public speeches, academic lectures, webinars, class presentations, wedding toasts, courtroom arguments, sermons, product demonstrations, and business presentations [6–8], and because basic questions remain about how to present effectively. Should we present with or without presentation software? If we should present with software, which software? We examined PowerPoint and Prezi because they are popular and psychologically interesting alternatives: Whereas PowerPoint’s linear slide format might reduce cognitive load, focus attention, and promote logical analysis, Prezi’s map-like canvas format and heavy reliance on animation (see the Background section and https://prezi.com for examples) might facilitate visuospatial processing, conceptual understanding, and narrative storytelling.

## Background

To inform the present research, we explore the methodological challenges of media research and review past research on presentation formats.

### Methodological challenges of media research

To research the efficacy of different communication formats fairly and accurately, one must overcome two stubborn methodological challenges. First, because correlation is not causation and the variables that underlie media usage are heavily confounded, such research requires true experimentation. To study whether a blended learning “flipped classroom” is a more effective instructional medium than traditional lecturing, for example, researchers gain little insight by comparing outcomes for students who enroll in one type of course versus the other. To control for audience (in this case, student) self-selection effects, researchers need to 1) randomly assign audience members to different communication conditions (in this case, pedagogies) or 2) manipulate format within participants. Moreover, the same methodological controls need to be applied to presenters (in this case, instructors). Instructors who choose to teach with emerging, innovative methods probably differ in numerous other respects (e.g., motivation) from those who teach with more traditional methods. If students assigned randomly to a flipped classroom format perform better than those assigned randomly to a traditional classroom format, we risk drawing inferences about confounds instead of causes unless instructors are also assigned randomly to instructional media. To make strong, accurate inferences, therefore, researchers interested in communication must control for audience and presenter self-selection effects. Such control introduces new complexities; when randomly assigning presenters to formats, for example, one must ensure that all presenters receive sufficient training in the relevant format. Moreover, such control is often cumbersome, sometimes impractical, and occasionally unethical (e.g., randomly assigning students in actual courses to hypothetically worse instructional conditions). But there are no adequate methodological substitutes for proper experimental control.

A second thorny methodological challenge inherent in conducting media research concerns how to draw general inferences about formats instead of specific inferences about exemplars of those formats. For example, if one advertising expert is assigned randomly to design a print ad and another expert a television ad—and a hundred consumers are assigned randomly to view the television or print ad—can we actually infer anything about print versus television ads in general when the two groups of consumers behave differently? Arguably not, because such a finding is just as easily explained by other (confounding) differences between the ads or their creators (e.g., ratio of print to graphics, which sorts of people—if any—are shown, and so forth). In other words, even with proper random assignment, researchers who intend to study different *forms* of communication risk merely studying different *instances* of communication. Statistically speaking, one should assume a random not fixed effect of the communication objects of interest (e.g., presentations, lectures, advertisements). To overcome this challenge and draw generalizable inferences, one must (at the very least) sample a sufficiently large set of examples within each medium.

### Research on presentation software

#### Methodological shortcomings

Considerable research has been conducted on how different presentation formats (particularly PowerPoint) convey information (for review, see [9]). However, much of this research is anecdotal or based on case studies. For example, Tufte [10] claims that PowerPoint’s default settings lead presenters to create bulleted lists and vacuous graphs that abbreviate arguments and fragment thought. And Kjeldsen [11] used Al Gore’s TED talk on climate change as a positive example of how visuals can be used to effectively convey evidence and enhance verbal communication.

Research that goes beyond mere anecdote or case study is plagued by the aforementioned methodological shortcomings: failure to control for audience self-selection effects (71% of studies), failure to control for presenter self-selection effects (100% of studies), and a problematic assumption of fixed effects across content and presenters (91% of studies). As is evident in Table 1, no studies overcame two of these shortcomings, let alone all three. For example, in one of the most heavily-cited publications on this topic Szabo and Hasting [12] investigated the efficacy of PowerPoint in undergraduate education. In the first study, they examined whether students who received lectures with PowerPoint performed better on a test than students who received traditional lectures. Students were not assigned randomly to lecture conditions, however; rather, the comparison was across time, between two cohorts of students enrolled in different iterations of the same course. Any observed outcome difference could have been caused by student or instructor variables (e.g., preparedness), not lecture format. The fact that no such differences were found does not obviate this concern: Such differences may in fact have been present, but were overshadowed by confounding characteristics of students or instructors. In the second study, the authors varied presentation format within the same cohort of students, but confounded format with order, time, content, and performance measure: student performance was compared between lectures on different days, on different topics, and using different tests. As the authors themselves note, the observed differences may have had nothing to do with PowerPoint. In the third study, they counterbalanced lecture order and content; some students received a PowerPoint lecture first and others a traditional lecture first, and the same topics were presented in both formats. However, students were assigned to conditions based on their course enrollment, not randomly, but more importantly the study included only four presentations, all by one presenter. Any advantages of the two PowerPoint lectures (none were found) might have been particular to those instances or that presenter and not representative of the format more generally.

**Table 1:** Findings and flaws of research into presentation software.

	**PPT vs Traditional**		
Outcome Variable	PPT > Trad	Null	PPT < Trad
Performance	9 of 28 (32%)	17 of 28 (61%)	2 of 28 (7%)
Perception	21 of 26 (81%)	3 of 26 (12%)	2 of 26 (8%)
Persuasion	2 of 2 (100%)	0 of 2 (100%)	0 of 2 (100%)
	**PPT Only**		
	Positive	Neutral	Negative
Perception	7 of 7 (100%)	0 of 7 (0%)	0 of 7 (0%)
	**Prezi vs PPT**		
	Prezi > PPT	Null	Prezi < PPT
Performance	0 of 2 (0%)	2 of 2 (100%)	0 of 2 (0%)
Perception	1 of 1 (100%)	0 of 1 (0%)	0 of 1 (0%)
	Methodological Shortcomings		
Did not control for audience self-selection effects	47 of 66 (71%)		
Did not control for presenter self-selection effects	66 of 66 (100%)		
Assumed fixed presenter or presentation effects	60 of 66 (91%)		
Studied researchers' own presentations or students	54 of 66 (82%)		

Note: "PPT Only" studies investigated whether participants had positive, neutral, or negative perceptions of PowerPoint and did not explicitly contrast PowerPoint with other presentation formats. All studies were coded by two authors (STM and ST), with any discrepancies (4%) resolved by discussion. To determine whether researchers studied their own presentations or students, we sometimes made inferences based on researchers' published academic affiliations, appointments, and disciplines, as well as other online information (e.g., curricula vitae).

Most studies—even those that control experimentally for audience self-selection—relied on only a single self-selected presenter, and some relied on only one presentation per format. In one study ([13]: Experiment 1), for example, one of the authors varied the format of his lecture instruction randomly across the semester, using transparences or PowerPoint slides. In another study [14], students who were enrolled in one of the authors’ courses were assigned randomly to a PowerPoint or Prezi e-lecture that contained identical audio narration and written text. In a third study [15], one of the researchers gave the same lecture over the course of the year to rotating medical students, using PowerPoint on odd months and overhead slides on even months. What reason is there to think that we can make general claims about presentation format based on studies of single lectures or single presenters? That is, how can we reasonably assume fixed as opposed to random effects? If the use of presentation software does meaningfully influence student learning or experience, surely that effect is not constant across all presenters or presentations—some instructors use it more effectively than others, and within any format some presentations are more effective than others (see [16]). And how can we assume that presenters who select both the content and format of their presentations are not designing them in ways that favor one format over another?

Research on the efficacy of presentation software has numerous other flaws, most notably the failure to control for experimenter effects or demand characteristics. In 82% of studies we identified, for example, the researchers investigated their own instruction and studied their own students. It is difficult to imagine that one would make these instructional and research efforts (e.g., creating new course material, conducting a field experiment) without a strong belief in the efficacy of one format over the other, and it is plausible (if not likely) that such beliefs would influence students or confound instructional format with instructional effort and enthusiasm.

Another common issue is the confounding of lecture format with access to study materials—in studies that contrast PowerPoint with traditional lecturing (e.g., [17–19]), students in the PowerPoint condition (but not the control condition) sometimes have access to PowerPoint slides as study material. This access could bias student motivation, behavior (e.g., attendance), course satisfaction, and performance (see [20]).

#### PowerPoint: Performance, perception, and persuasion

Despite their methodological shortcomings, what are the findings of this research literature? The majority of studies examined the use of PowerPoint in higher education and measured both objective and subjective outcomes (see Table 1). They typically involved students enrolled in one or more of the researchers’ courses, and contrasted the efficacy of lectures (or whole lecture courses) that used PowerPoint with those that used a more traditional technology (e.g., blackboards, overhead projectors). In terms of student performance, their findings were notably mixed: Of the 28 studies we identified, 17 found no effect of PowerPoint lectures relative to traditional lectures ([12]: Experiments 1,3; [13,15,21–33]), 9 found a performance benefit of PowerPoint over traditional instruction ([12]: Experiment 2; [17–19,34–38]), and 2 found a performance benefit of traditional over PowerPoint instruction [39,40].

There is near consensus in the literature, however, when it comes student perception: Of the 26 studies we identified, 21 found that students preferred PowerPoint over traditional instruction ([12]: Experiment 1; [13,17–19,21,23,25,26,28,29,31–33,35,39,41–45]), 2 found that students preferred traditional over PowerPoint instruction [40,46], and 3 other studies found no preference for one or the other formats [15,22,37]. As one example, Tang and Austin [45] surveyed 215 undergraduates in business courses about their general perceptions of different lecture formats; on measures of enjoyment, learning, motivation, and career relevance, they found that students rated lectures with PowerPoint slides more favorably than lectures with overheads or without visual aids. An additional 7 studies did not contrast student perceptions of PowerPoint with another technology—they simply surveyed students about PowerPoint; these studies all found that students had, on average, favorable impressions of PowerPoint-based instruction [36,47–52].

In addition to these studies of how presentation software impacts student performance and perception, two studies examined PowerPoint‘s impact on audience persuasion. Guadagno, Sundie, Hardison, and Cialdini [53] argue that we heuristically use a presentation’s format to evaluate its content, particularly when we lack the expertise to evaluate the content on its merits. To test this hypothesis, they presented undergraduates with key statistics about a university football recruit and asked them to evaluate the recruit’s career prospects. The same statistics were presented in one of three formats: a written summary, a graphical summary via printed-out PowerPoint slides, or a graphical summary via animated PowerPoint slides (self-advanced by the participant). Participants shown the computer-based PowerPoint presentation tended to rate the recruit more positively than other participants, and there was some evidence that this effect was more pronounced for football novices than for experts. The findings of this study suggest that some presentation formats may be more persuasive than others, perhaps because audience members conflate a sophisticated medium with a sophisticated message.

In the second study to examine the impact of PowerPoint on persuasion, Park and Feigenson [54] examined the impact of video-recorded presentations on mock juror decision-making. Participants were more persuaded by attorneys on either side of a liability case when the attorney used PowerPoint slides as opposed to merely oral argument. They also remembered more details from PowerPoint than oral presentations, and evaluated both attorneys as more persuasive, competent, credible, and prepared when they presented with PowerPoint. Based on mediation analyses, the researchers argue that the decision-making benefit of PowerPoint results from both deliberative and heuristic processing (“slow” and “fast” thinking, respectively, see [55]).

Both of these studies, however, share the methodological limitations of the educational research on PowerPoint. The first study [53] used only one PowerPoint presentation, and the second [54] used only two. The presentations used were not selected at random from a larger stimulus pool but instead were created by researchers who hypothesized that PowerPoint would enhance presentations. But even if the presentations had been sampled randomly, the sample is too small to allow one to generalize to a broader population. In studying performance, perception, or persuasion, one cannot reasonably assume that all presentation effects are equal.

#### Prezi: A zoomable user interface

Released in 2009, Prezi has received generally favorable reviews by researchers, educators, and professional critics [56–60]. With a purported 75 million users worldwide, it is increasingly popular but still an order of magnitude less so than PowerPoint (with as many as one billion users; [61]). Like PowerPoint and other slideware, Prezi allows users to arrange images, graphics, text, audio, video and animations, and to present them alongside aural narration to an in-person or remote audience. In contrast to PowerPoint and other slideware in which users create presentations as a deck of slides, Prezi users create presentations on a single visuospatial canvas. In this regard, Prezi is much like a blackboard and chalk. But unlike a physical blackboard, the Prezi canvas is infinite (cf. [62]) and zoomable: in designing presentations, users can infinitely expand the size of their canvas and can zoom in or out. When presenting, users define paths to navigate their audience through the map-like presentation, zooming and panning from a fixed-angle overhead view.

Like Google Maps or modern touchscreens, Prezi is an example of what scholars of human-computer interaction label a zoomable user interface (ZUI). These interfaces are defined by two features: They present information in a theoretically infinite two-dimensional space (i.e., an infinite canvas) and they enable users to animate this virtual space through panning and zooming. Some of the original ZUIs were used to visualize history, navigate file systems, browse images, and—in the Prezi predecessor CounterPoint—create presentations [63, 64].

As communication and visualization tools, ZUIs in general and Prezi in particular are interesting psychologically for several reasons. First, they may take advantage of our mental and neural architecture, specifically the fact that we process information through dissociable visual and spatial systems. Whereas the so-called “ventral” visual system in the brain processes information such as shape and color, the “dorsal” spatial system processes information such as location and distance [65–68]. When working in concert, these systems result in vastly better memory and comprehension than when they work in isolation. For example, in the classic “method of loci” individuals visualize objects in specific locations; when later trying to recall the objects, they visualize navigating through the space, “seeing” each object in turn. This method typically doubles retention, compared to other ways of trying to memorize objects [69, 70]. Similarly, in research on note-taking, students learned more when they used spatial methods than when they used linear methods (e.g., [71]). Mayer’s multimedia learning principles and evidence in their favor also highlight the importance of spatial contiguity [72].

Thus, by encouraging users to visualize and process information spatially, ZUIs such as Prezi may confer an advantage over traditional tools such as PowerPoint that do not encourage such visuospatial integration. As Good and Bederson [64] write: “Because they employ a metaphor based on physical space and navigation, ZUIs offer an additional avenue for exploring the utilization of human spatial abilities during a presentation.”

Furthermore, ZUIs may encourage a particularly efficacious type of spatial processing, namely graphical processing. In graphical processing, digital objects (or groups of objects) are not just arranged in space, they are arranged or connected in a way makes their interrelationships explicit. Randomly placing animal stickers on a blank page, for example, engages mere spatial processing; drawing connecting lines between animals of the same genus or arranging the animals into a phylogenetic tree, however, engages graphical processing. Because ZUIs force users to “see the big picture,” they may prompt deeper processing than software that segments content into separate spatial canvases. By facilitating such processing, ZUIs may leverage the same learning benefits of concept maps and other graphical organizers, which have been studied extensively. For example, in their meta-analysis of the use of concept maps in education, Nesbit and Adesope [73] found that these graphical representations (especially when animated) were more effective than texts, lists, and outlines. By requiring one to organize the whole presentation on a single canvas instead of a slide deck, therefore, Prezi may prompt presenters (and their audiences) to connect component ideas with each other, contextualize them in a larger narrative, and remember, understand, and appreciate this larger narrative. Slideware, on the other hand, may do just the opposite:

PowerPoint favours information that can be displayed on a single projected 4:3 rectangle. Knowledge that requires more space is disadvantaged … How to include a story on a slide? Distributing the associated text over several slides literally breaks it into fragments, disturbing its natural cohesion and thus coherence … PowerPoint renders obsolete some complex narrative and data forms in favour of those that are easily abbreviated or otherwise lend themselves to display on a series of slides [74] (p399)

Of course these arguments are speculative, and one can also speculate on the psychological costs of ZUI or benefits of standard slideware. Perhaps PowerPoint does confer some of same spatial processing benefits of Prezi—after all, slides are spatial canvases, and they must be arranged to form a narrative—but in a way that better manages the limited attentional resources of the presenter or audience. Our point here is simply that Prezi, as a ZUI presentation tool, offers a psychologically interesting alternative to standard deck-based slideware, with a range of possible advantages that could be explored empirically to discover the psychological mechanisms of effective communication.

Like the PowerPoint literature, most of the published literature on Prezi is limited to observational reports or case studies. Brock and Brodahl [75] evaluated Prezi favorably based on their review and students’ ratings of course presentations. Conboy, Fletcher, Russell, and Wilson [76] interviewed 6 undergraduates and 3 staff members about their experiences with Prezi in lecture instruction and reported generally positive experiences. Masood and Othman [77] measured the eye movements and subjective judgments of ten participants who viewed a single Prezi presentation; participants attended to the presentation’s text more than to its other components (e.g., images, headings), and favorably judged the presentation. Ballentine [78] assigned students to use Prezi to design text adventure games and reported benefits of using the medium. Two other studies [79, 80] surveyed college students about their course experiences with Prezi, and both reported similarly positive perceptions.

All of these studies, however, suffer from major demand characteristics, due to the fact that the researchers observed or asked leading questions of their own students about their own instruction (e.g., “Do you find lectures delivered with Prezi more engaging then[sic] other lectures?”, from [79]). Moreover, all suffer from the methodological limitations discussed earlier.

Other literature that addresses Prezi is purely theoretical and speculative: In discussing the pedagogical implications of various presentation software, Harris [81] mostly just describes Prezi’s features, but does suggest that some of these features provide useful visual metaphors (e.g., zooming in to demonstrate otherwise hidden realities). Bean [82] offers a particularly compelling analysis of PowerPoint and Prezi’s histories, user interfaces, and visual metaphors, and argues that Prezi is the optimal tool for presenting certain types of information (e.g., wireflow diagrams).

The experimental literature on Prezi is limited to three published studies. Castelyn, Mottart and Valcke [14] investigated whether a Prezi e-lecture with graphic organizers (e.g., concepts maps) was more effective than a PowerPoint e-lecture without graphic organizers. Claiming that Prezi encourages the use of graphic organizers, they purposefully confounded the type of presentation software with the presence of graphic organizers. Undergraduates randomly assigned to the different e-lectures did not differ in their knowledge or self-efficacy gains, but did prefer the graphically-organized Prezi lecture over the PowerPoint control lecture. In a follow-up study [83], the same researchers assigned undergraduates to create Prezi presentations that did or did not use graphic organizers, and found no effects of this manipulation on students’ self-reported motivation or self-efficacy. Chou, Chang, and Lu [24] compared the effects of Prezi, PowerPoint and traditional blackboard instruction on 5^th^ graders’ learning of geography. Whereas the Prezi group performed better than the control group (which received blackboard instruction) in formative quizzes and a summative test, the PowerPoint group did not; however, on a delayed summative test, both Prezi and PowerPoint students performed better than those in the control group. In direct comparisons of PowerPoint and Prezi, there were no differences in any of the learning measures. Taken together, the studies are not just limited in number: They present uncompelling findings and suffer from the same methodological shortcomings of the PowerPoint research.

### The current study

In short, the extant literature does not clarify whether presenters should present with or without visual aids—and, if the latter, whether they should use standard deck-based slideware such as PowerPoint or a ZUI such as Prezi. One of the reasons why these basic questions remain unanswered is the methodological challenges inherent in comparing different presentation formats. We designed the current study to overcome these challenges.

To control for individual differences among presenters, we randomly assigned presenters to different presentation conditions. To control for individual differences among audience members, we used a counterbalanced, within-participants design for the first experiment, and between-participants random assignment in the second experiment. And to draw general inferences about the impact of presentation format—instead of specific inferences about particular presenters or presentations—we sampled from a large number of presentations, each created by a different presenter. Our methods have their own challenges, such as recruiting participants sufficiently trained in all presentation methods, allowing presenters adequate preparation time and context, approximating the psychological conditions of real-world presentations, and measuring the “signal” of presentation format among the added “noise” of so many presenters and presentations. In addition, the studies had to be double-blind: Neither presenters nor audience members could be aware of any hypotheses, and had to be free from any sorts of confirmation bias conveyed by the investigators.

To focus on presentations as a form of presenter-audience communication and limit the number of confounded variables, we purposefully controlled for other possible impacts of presentation software on professional practices or outcomes, including 1) the use of presentation artifacts (e.g., PowerPoint files, printed-out slides, online Prezis), and 2) facilitated collaboration among presentation designers. Unlike other research (e.g., [32, 33]) we did allow for the possibility that presentation format not only affects how audiences perceive presentations, but also how presenters design or deliver them (e.g., by increasing their conceptual understanding of the topic, or decreasing their cognitive load during live narration; cf. [84]). In other words, presentation technologies might affect the cognition of both the audience and the presenter, so we designed the present studies to accommodate both sets of mechanisms.

To maximize the real-world relevance of this research, we relied on multimedia case materials from Harvard Business School [85]; these materials recreate the actual professional circumstances in which presentations are typically used. Because presentations are designed commonly both to inform and convince audiences, we examine outcome measures of learning as well as persuasion. And to minimize demand characteristics, we avoided the typical flaws of existing research (e.g., researcher-designed presentations, the researchers’ students as research participants) and adopted several countermeasures (e.g., recruitment language and participant instructions that obscured the research hypotheses, between-participant manipulation).

We adopted a two-phased approach in this research. In the first phase, participants with sufficient experience in oral, PowerPoint, and Prezi presentation formats were randomly assigned to create a presentation in one of those formats. We provided the necessary context, instruction, and time to create a short but realistic presentation. Participants then presented live to an actual audience, who judged each presentation’s efficacy. In the second phase, recorded versions of these presentations were presented to a larger online audience, affording us greater statistical power and allowing us to measure the impact of presentation format on decision-making and learning.

## Experiment 1

### Methods

#### Participants

We recruited presenter participants via online postings (on Craigslist, the Harvard Psychology Study Pool, the Harvard Decision Science Lab Study Pool), email solicitations to the local Prezi community, and campus flyers. To create the fairest comparison between PowerPoint and Prezi, we recruited individuals who “have expertise in using both PowerPoint and Prezi presentation software.” Interested individuals were directed to a prescreening survey in which they reported their experience with and preference for giving different types of presentations. Only individuals who reported that they were “not at all experienced” with PowerPoint, Prezi or giving oral presentations were excluded from research participation. Out of the 681 respondents who completed the prescreening survey, 456 of them were eligible and invited to sign up for an available timeslot. Out of this group, 146 individuals—105 from the Harvard study pools, 33 from Craigslist, and 8 from the Prezi community—participated as presenters in the study and were compensated $40 for approximately two hours of their time. There were no significant differences between the three presentation groups on any demographics variables.

We also recruited 153 audience participants from the Harvard Decision Science Lab Study Pool and Craigslist using the following announcement:

Do you use Skype? Does your computer have a large screen (13 inches or larger)? If so, you may be eligible to participate in a 45 minute long online study. In this study, you will watch professional presentations over Skype from home on your personal computer.

Anyone who responded to the recruitment notice was eligible, provided that they were available during one of the prescheduled testing sessions. Audience participants were compensated $10 for approximately 45 minutes of their time. Table 2 presents demographic information for the presenter and audience participants. This study was approved by the Harvard Committee on the Use of Human Subjects (Study #IRB14-1427), and all participants in both experiments provided written consent.

**Table 2:** Participant demographics for Experiment 1.

		Presenter	Audience
Gender identity	Female	63	106
	Male	83	45
	Other	0	1
Mean age (in years)		23.6	27.4
SD age (in years)		6.0	9.4
English fluency	Native	107	105
	Non-native, fluent	39	45
	Non-native, non-fluent	0	2
Occupation	Student	102	102
	Other	37	43
	Unemployed	3	7
Education level	High school	35	10
	Vocational school	0	1
	College (some credit)	52	33
	College (completed)	38	58
	Graduate or professional school	19	50

Note: Unless otherwise noted, numbers refer to the number of participants in each category. Two presenter participants did not report their educational level, and four did not report their occupation. Demographic data from one audience participant is missing due to a coding error.

#### Presenter procedure

Presenter participants completed a survey remotely before attending the in-person, group sessions with other participants. In the online pre-survey, presenters first answered basic demographic questions (gender, age, education level, English fluency, and occupation). Next, they answered questions about their prior experience with, opinions about, and understanding of the different presentation formats (oral, Prezi, and PowerPoint). This section was prefaced with the following note:

A note on language: When we use the term "presentation," we mean a formal, planned, and oral presentation of any duration, including a public speech, an academic lecture, a webinar, a class presentation, a wedding toast, a sermon, a product demonstration, a business presentation, and so on. Examples of things we do NOT mean are: a theatrical performance, an impromptu toast at dinner, and any presentation with no audience. When we say PowerPoint presentations, we mean presentations that were made using Microsoft PowerPoint, not other software such as Apple's Keynote. When we say Prezi presentations, we mean presentations that were made using Prezi presentation software. Also, when we refer to "oral presentation", we mean a presentation that is only spoken and does not include any visual aids or the use of presentation software.

Participants were asked the following questions for each type of presentation:

- How experienced are you at making the following types of presentations? [5-level rating]
- When you give a presentation, how effective are the following types of presentations for you? [5-level rating, with “not applicable” option]
- When somebody else gives a presentation, how effective are the following types of presentations for you? [5-level rating, with “not applicable” option]
- How difficult is it for you to make the following types of presentations? [5-level rating, with “not applicable” option]
- In the last year, approximately how many of the following types of presentations did you make? [free response]
- In your lifetime, approximately how many of the following types of presentations have you made? [free response]
- For approximately how many years have you been making the following types of presentations? [free response]

As part of the expertise-related measures, we also asked the participants to identify the purported advantages and disadvantages of each presentation format, according to its proponents and critics, respectively. For PowerPoint and Prezi, we asked participants to identify whether or not it had particular functionalities (e.g., the capacity to record narration, create custom backgrounds, print handouts). Finally, participants viewed three sets of four short Prezi presentations and rank-ordered them from best to worst. In each set we manipulated a key dimension of Prezi effectiveness, according to its designers: the use of zooming, the connection of ideas, and the use of visual metaphor.

Presenter participants were tested in person at the Harvard Decision Science Lab, and randomly assigned to one of the three groups: Prezi, PowerPoint, or oral presentation. A total of 50 data collection sessions were held. In each session, there were typically three presenter participants (one for each presentation format); as a result of participants who failed to arrive or overbooking, there were ten sessions with only two presenters and six sessions with four presenters.

After providing informed consent, participants completed an online survey (in the lab) in which they rank-ordered three sets of recorded example PowerPoint and oral presentations. Identical in form to the example Prezi presentations they judged in the pre-survey, these short presentations were designed to assess their understanding of effective presentation design by manipulating a key aspect specific to each format. For PowerPoint presentations, we manipulated the use of text, use of extraneous “bells and whistles,” and graph design; for oral presentations, the three dimensions were verbal behavior, nonverbal behavior (other than eye contact), and eye contact. In selecting these dimensions (and those for Prezi), we consulted with a variety of experts, including software designers, speaking coaches, and researchers.

Next, presenters were shown material from a multimedia case created for and used by the Harvard Business School. Specifically, they were told the following (the company featured in the business case will be referred to anonymously here as “Company X” to respect their contractual agreement with the school):

For the next two hours, you are going to pretend to be the chief marketing officer of i-Mart, a large chain of retail stores. i-Mart recently made an offer to [Company X] to sell their products in i-Mart stores. Your boss, the CEO of i-Mart, has asked you to make a presentation to [Company X]’s leadership that persuades them to accept i-Mart’s offer. In your presentation, you will need to argue that accepting i-Mart’s offer is in [Company X]’s strategic interests, and address any concerns they may have about how accepting the offer might affect their corporate identity.

As a participant in this study, your primary job today is to prepare and then deliver this presentation. The presentation will be very short (less than 5 minutes) and made live (via Skype) to an audience of participants who are playing the part of [Company X] executives. Before you start planning your presentation, you will first learn more about [Company X] and how they’re thinking about i-Mart’s offer.

On their own computer workstation, participants studied the multimedia case for 30 minutes and were invited to take notes on blank paper provided for them. The multimedia case material included video and textual descriptions of Company’s X’s corporate culture, business model, and constituent communities.

Following this study period, participants were given 45 minutes to create a presentation in one of three randomly assigned presentation formats: PowerPoint, Prezi, or oral. To assist participants in the PowerPoint and Prezi conditions, we provided them with a set of digital artifacts including text, data, and graphics related to the case. Participants were not told that other participants were asked to present in different formats, and the workstations were separated from each other to prevent participants from discovering this manipulation.

After this preparation period, participants were taken individually (in a counterbalanced order) to another room to present to a live audience via Skype. For PowerPoint and Prezi presentations, we shared each participant’s presentation with the audience via screen sharing; thus they viewed both the presenter and the presentation. For those presenters who consented, we also recorded their presentations for future research purposes. After making their presentations, presenters completed a final survey about their presentation (e.g., “How convincing do you think your presentation will be to [Company X’s] board members”), the corporate scenario (e.g., What do you think [Company X] should do?”), and their presentation format (e.g., “How likely are you to recommend the presentation tool or presentation format you used to others to make professional presentations?”).

#### Audience procedure

Audience participants completed the entire experiment remotely and online. Their participation was scheduled for the end of the presenter sessions so that the in-lab presenters could present live to a remote audience via Skype. We recruited between three and six audience participants per session, although participants who failed to arrive or Skype connectivity issues resulted in some sessions with only one or two audience participants: Five sessions had one participant, twelve sessions had two participants, sixteen sessions had three participants, eleven sessions had four participants, four sessions had five participants, and two sessions had six participants.

Individuals who responded to the recruitment notice completed a consent form and three online surveys prior to their scheduled Skype session. The first survey was a slightly modified form of the presenter pre-survey (demographics, background on presentation formats, rank-ordering of example Prezis) in which they also scheduled their Skype session. In the second survey, audience participants were told that they were “going to play the role of a corporate executive listening to several short business presentations,” and that their task was “to evaluate the quality of these presentations, each made by another participant engaged in a similar role-playing scenario.” They were then shown a brief video and textual description of the fictionalized corporate scenario (an abridged version of what presenter participants studied), and told the following:

You are a board member for [Company X], an innovative clothing company. Another company, i-Mart, wants to sell [Company Y’s products] in its stores. You and your fellow board members must decide whether or not to accept i-Mart's offer.

And in the third survey they rank-ordered the three sets of recorded example PowerPoint and oral presentations.

At the time of the scheduled session, the audience participants logged into Skype using a generic account provided by the research team, and were instructed to turn on their webcams and put on headphones. Once the first presenter participant was ready to present, the experimenter initiated the group Skype call, confirmed that the software was functioning properly, invited the presenter into the room to begin, left the room before the start of the presentation, monitored the presentation remotely via a closed-circuit video feed, and re-entered the room at the presentation’s conclusion. For Prezi and PowerPoint presentations, Skype’s built-in screen-sharing function was used to share the visual component of the presentation; audience participants viewing these presentations were instructed to use the split-screen view, with windows of equal size showing the presenter and the accompanying visuals.

Immediately after viewing each presentation, participants evaluated it via an online survey. They rated each presentation on how organized, engaging, realistic, persuasive, and effective it was using a five-level scale with response options of *not at all*, *slightly*, *somewhat*, *very*, and *extremely*. They were also invited to offer feedback to the presenter on how the presentation could be improved. After the final presentation, participants rank-ordered the presentations on the same dimensions (e.g., effectiveness, persuasiveness). Halfway through the experiment we added a final question in which we asked participants to rank-order PowerPoint, Prezi, and oral presentation formats “in terms of their general effectiveness, ignoring how well individual presenters (including today's) use that format,” and to explain their rank-ordering.

### Results

#### Prior experience and pre-existing beliefs

Participants’ prior experience with and pre-existing beliefs about each presentation format provide a baseline that informs the research findings. If presenter participants had more experience with and more positive beliefs about one format than the others—and those assigned to that format induced more positive assessments from the audience members than did those assigned to the other formats—then the results are less compelling than if there was no correlation between these baseline measures and the experimental outcomes. The same applies to audience participants: Are they merely judging presentations according to their initial biases? Conversely, the results are most compelling if there is a negative association between the baseline measures and the experimental findings. For this reason—and to check that presenters assigned to the different formats did not happen to differ in these baseline measures—we analyzed participants’ prior experience with and pre-existing beliefs about PowerPoint, Prezi, and oral presentation formats.

Both audience and presenter participants were least experienced with Prezi and most experienced with oral presentations. At the outset, they rated PowerPoint as the most effective and easiest to use to present material and Prezi as the least effective and most difficult to use to present. For watching presentations, audience participants rated PowerPoint most effective and oral presentations least effective, but rated Prezi as more enjoyable than other formats. For watching presentations, presenter participants did not find any format more effective than the others. Table 3 presents full descriptive and inferential statistics for all self-reported measures of prior experience with and preexisting beliefs about Prezi, PowerPoint, and oral presentations.

**Table 3:** Prior experience and pre-existing beliefs of Experiment 1 participants.

	Means						
	Prezi	PPT	Oral	*F*	N	*P*	post hoc
	Presenter participants						
Presenter experience (rating)	2.95	4.13	3.96	156.1	142	<.00001	PPT > Oral > Prezi
Presenter effectiveness (rating)	3.45	3.96	3.87	20.1	139	0.001	PPT > Oral > Prezi
Audience effectiveness (rating)	3.80	3.87	3.70	1.9	139	0.16	--
Presenter difficulty (rating)	3.53	2.33	2.85	31.5	137	<.00001	Prezi > Oral > PPT
Presentations made last year (#)	1.45	8.46	8.20	20.2	142	<.00001	PPT = Oral> Prezi
Presentations made in lifetime (#)	6.62	48.7	58.1	46.0	142	<.00001	PPT = Oral> Prezi
Presenter experience (years)	2.45	8.83	10.2	293	142	<.00001	Oral > PPT > Prezi
	Audience participants						
Presenter experience (rating)	1.99	3.74	3.65	245.8	152	<.00001	PPT = Oral > Prezi
Presenter effectiveness (rating)	3.26	3.92	3.77	15.7	98	<.00001	PPT > Oral > Prezi
Audience effectiveness (rating)	3.68	3.82	3.56	2.7	117	0.07	PPT > Oral
Audience enjoyableness (rating)	3.68	3.35	3.35	4.7	117	0.01	Prezi > PPT = Oral
Presenter difficulty (rating)	2.71	1.89	2.27	30.1	125	<.00001	Prezi > Oral > PPT
Presentations watched last year (#)	2.4	30.4	27.1	35.5	152	<.00001	PPT = Oral > Prezi
Presentations watched in lifetime (#)	10.1	469.0	1127.6	10.4	148	0.001	Oral > PPT > Prezi
Audience experience (years)	2.1	11.5	16.3	287.1	151	<.00001	Oral > PPT > Prezi

Note: *F*-statistics and *p*-values are from one-way ANOVAs with Presentation Format as the within-participant factor; for items with significant non-sphericity, we adjusted the dfs as necessary and confirmed the results with non-parametric Friedman tests. For rating items in which participants could indicate “Not applicable (I have never seen/given this type of presentation)”, the reported means, *F*-statistics, sample sizes, and *p*-values include only data from participants who did not select this response option; no presenter participants selected this response option. For rating items, post-hoc inferences are drawn from Fisher’s LSD tests (using all available data); for frequency items, post-hoc inferences are drawn from non-parametric Wilcoxon signed-rank tests due the skewed distributions and presence of outliers.

Presenters assigned to different formats did not differ in their experience with or pre-existing beliefs about presentations formats. They also did not differ in how well they identified the purported advantages and disadvantages of each presentation format, how well they identified the software features of PowerPoint and Prezi, or how accurately they could identify effective presentations of each format.

#### Audience ratings

In term of their prior experience with and pre-existing beliefs about presentation formats, both audience and presenter participants were biased in favor of oral and PowerPoint presentations and against Prezi. After presenters were randomly assigned to these different formats, how did the audience evaluate their presentations?

In examining how presentation format affected the audience’s ratings of the presentations, two complications arose. First, sessions with two presentations were missing one presentation format, and sessions with four presentations had two presentations of the same format. To address this complexity we only conducted pairwise comparisons of different formats (e.g., PPT versus oral) instead of omnibus tests, and—for those sessions with four presentations—we averaged ratings for the two same-format presentations. To be certain that the differing number of presentations per session did not somehow bias the results even after adopting these measures, we also conducted an analysis on the subset of sessions that had exactly three presentations.

Second, the number of audience participants per session ranged from one to six. In calculating descriptive statistics, some sessions would be weighted more heavily than others unless ratings were first averaged across participants within the same session, then averaged across sessions. In calculating inferential statistics, averaging across ratings from different participants within the same session who received presentations in the same format was necessary to ensure that the sampling units were independent of each other, an assumption of all parametric and most nonparametric tests. In other words, for both descriptive and inferential statistics, we treated session (instead of participant) as the sampling unit.

As an empirical matter, this multi-step averaging—within participants across identical presentation formats, then across participants within the same session—had little impact on the condition means (i.e., the average ratings of PowerPoint, Prezi, or oral presentations on each dimension). Compared to the simplest, raw averaging of all ratings in one step, the maximum absolute difference between these two sets of means was .07 (on a 1–5 scale) and the mean absolute difference was .04.

**Fig 1:**
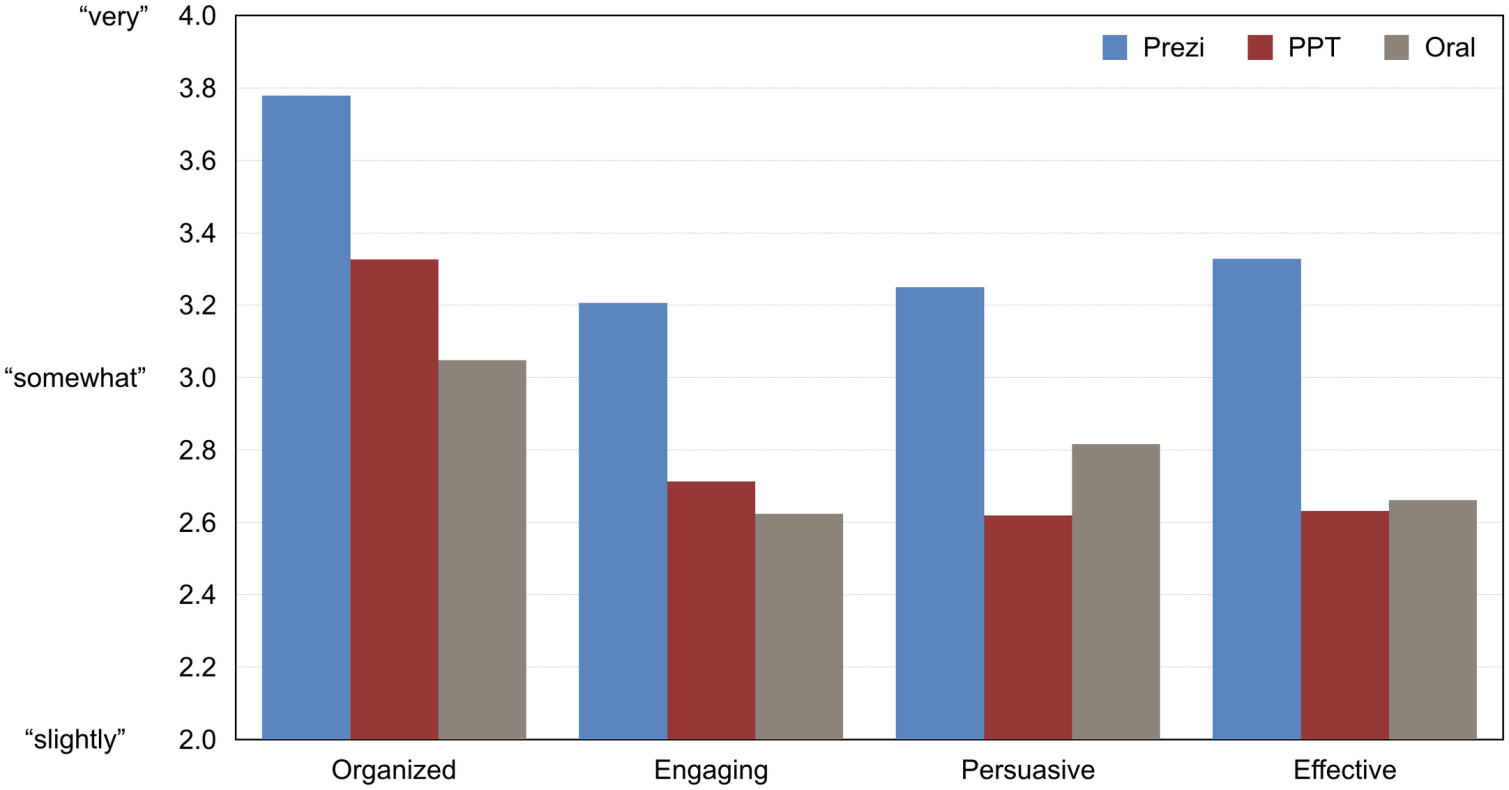
Experiment 1 audience ratings. Audience members rated presentations on each dimension on a 5-level scale (1 = “not at all,” 5 = “extremely”). The figure shows session-level means from all available data, including those from sessions with two or four presentations.

To test whether the presentations’ format affected their ratings, therefore, we conducted paired *t*-tests for each rating dimension, with presentation format as the repeated measure and mean session rating as the dependent variable. Because we conducted three tests for each dimension—pairing each format with every other—we controlled for multiple comparisons by dividing our significance threshold by the same factor (i.e., α = .05/3 = .017). Results revealed that presentation format influenced audience ratings. In particular, the audience rated Prezi presentations as significantly more organized, engaging, persuasive, and effective than both PowerPoint and oral presentations; on a five-level scale, the average participant rated Prezi presentations over half a level higher than other presentations. The audience did not rate PowerPoint presentations differently than oral presentations on any dimension. Table 4 and Fig 1 present these results.

**Table 4:** Descriptive and inferential statistics for Experiment 1 audience ratings.

	Prezi mean	PPT mean	Mean difference	*t*	*df*	*p*
Organized	3.78	3.36	0.42	3.22	43	0.002
Engaging	3.20	2.75	0.44	2.49	43	0.017
Persuasive	3.23	2.65	0.58	3.36	43	0.002
Effective	3.32	2.65	0.67	3.86	43	0.002
	Prezi mean	Oral mean	Mean difference	*t*	*df*	*p*
Organized	3.78	3.04	0.74	4.84	42	<.0001
Engaging	3.19	2.58	0.61	3.21	42	0.003
Persuasive	3.26	2.78	0.48	2.84	42	0.007
Effective	3.30	2.64	0.66	4.22	42	0.0001
	PPT mean	Oral mean	Mean difference	*t*	*df*	*p*
Organized	3.34	3.05	0.29	1.66	42	0.104
Engaging	2.68	2.60	0.08	0.42	42	0.677
Persuasive	2.59	2.84	-0.25	-1.34	42	0.187
Effective	2.63	2.66	-0.03	-0.14	42	0.887

Note: Because some sessions only had two presentations (i.e., Prezi and PPT, Prezi and Oral, or PPT and Oral), the mean ratings for the same format vary slightly across pairwise comparisons. The *p*-values are uncorrected for multiple comparisons; instead we used a corrected significance threshold of α = .05/3 = .017.

By limiting the analysis to the 34 sessions with exactly three presentations (one of each format), we could ensure that the sessions with two or four presentations did not somehow bias the results. Moreover, this procedure enabled us to conduct omnibus tests of presentation format for each rating dimension. These omnibus tests revealed significant effects for organization, *F*(2,66) = 12.9, *p* < .0001, engagement, *F*(2,66) = 4.6, *p* = .01, persuasion, *F*(2,66) = 3.9, *p* = .03, and effectiveness, *F*(2,66) = 7.2, *p* = .001. The results from post-hoc tests (Fisher’s LSD) aligned with the original pairwise comparisons: On all dimensions, the audience rated Prezi presentations higher than PowerPoint and oral presentations, *p*s < .05; PowerPoint and oral presentations were not rated differently on any dimension, *p*s>.05. (Note: All *p*-values for pairwise tests here and elsewhere are two-tailed.)

To explore whether the obtained results were somehow the result of demand characteristics, we analyzed ratings from only the first presentation in each session. This analysis yielded the same pattern of findings, with a to-be-expected reduction in statistical significance due to the loss of power. On all four dimensions, a one-way, independent-measures ANOVA yielded significant or marginally-significant results: organized, *F*(2,49) = 5.1, *p* = .01; engaging, *F*(2,49) = 2.5, *p* = .09; persuasive, *F*(2,49) = 2.6, *p* = .09; and effective, *F*(2,49) = 5.8, *p* = .006. In all cases, Prezi was rated higher than oral and PowerPoint presentations (post-hoc LSD *p*s ≤.08).

On average, the audience rated the presentations as realistic, with a modal rating of “very realistic.” Our intent in including this rating dimension was merely to verify that our experimental protocol resulted in realistic rather than contrived presentations; we therefore did not test for differences in these ratings as a function of group differences.

#### Audience rankings

As just noted, participants randomly assigned to present using Prezi were rated as giving more organized, engaging, persuasive, and effective presentations compared to those randomly assigned to the PowerPoint or oral presentation conditions. In addition, at the end of each session audience participants rank-ordered each type of presentation on the same dimensions used for the ratings. Here we ask: Did the audiences’ rank-orderings align with the ratings?

The same complexities with the ratings data—the variable number of conditions and audience participants per session—applied as well to the ranking data. We therefore adopted a similar analytic strategy, with one exception: we conducted non-parametric rather than parametric pairwise tests, given the rank-ordered nature of the raw data and distributional assumptions that underlie parametric tests.

**Fig 2:**
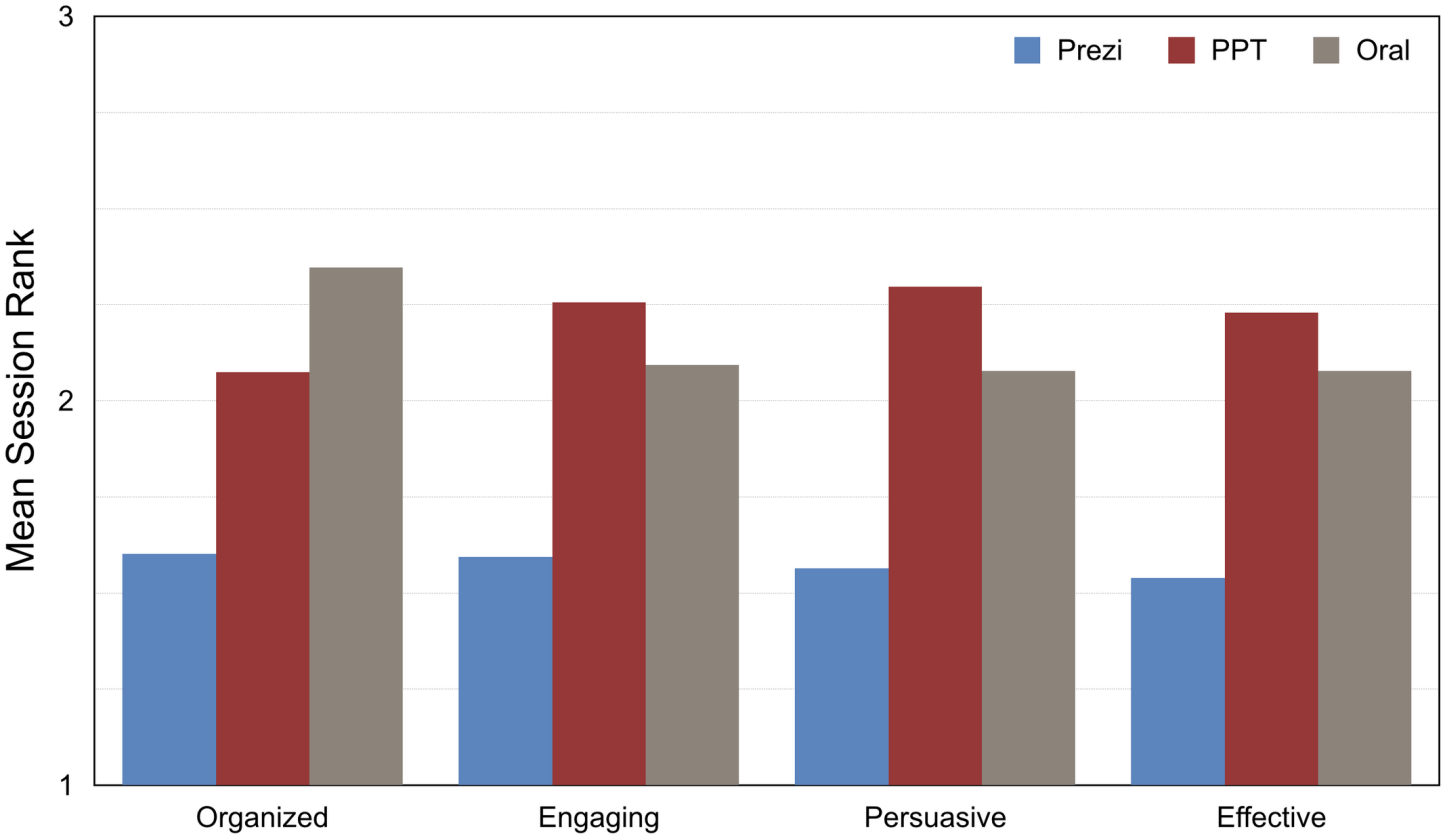
Experiment 1 audience rankings. Audience members ranked the presentations from best to worst, with lower ranks indicating better presentations. The figure shows session-level means from all available data, including those from sessions with two or four presentations.

Using the session-level mean ranks, we tested the effect of presentation format with three sets of Wilcoxon signed-rank tests. The results had the identical pattern as those from the ratings data: the audience rated Prezi presentations as significantly more organized, engaging, persuasive, and effective than both PowerPoint and oral presentation (all *p*s ≤ .006); the audience did not rate PowerPoint presentations differently than oral presentations on any dimension. Table 5 and Fig 2 present these results.

**Table 5:** Descriptive and inferential statistics for Experiment 1 audience rankings.

	Prezi mean	PPT mean	Mean difference	*Z*	*N*	*p*
Organized	1.64	2.13	-0.48	2.81	42	0.005
Engaging	1.59	2.29	-0.70	3.38	41	0.001
Persuasive	1.57	2.33	-0.77	3.78	42	0.0002
Effective	1.54	2.26	-0.72	3.33	41	0.001
	Prezi mean	Oral mean	Mean difference	*Z*	*N*	*p*
Organized	1.65	2.40	-0.74	4.12	41	0.0004
Engaging	1.63	2.16	-0.53	2.78	41	0.006
Persuasive	1.60	2.14	-0.54	2.63	41	0.009
Effective	1.57	2.27	-0.69	3.70	41	0.0002
	PPT mean	Oral mean	Mean difference	*Z*	*N*	*p*
Organized	2.09	2.37	-0.29	2.03	41	0.04
Engaging	2.29	2.15	0.14	-1.03	41	0.30
Persuasive	2.35	2.12	0.23	-1.43	41	0.15
Effective	2.25	2.24	0.01	-0.12	41	0.90

Note: Because some sessions only had two presentations (i.e., Prezi and PPT, Prezi and Oral, or PPT and Oral), the mean ratings for the same format vary slightly across pairwise comparisons. We did not add the rank-ordering until the third session, hence two sessions have missing data. All inferential statistics are from Wilcoxon signed-ranked tests. The p-values are uncorrected for multiple comparisons; instead we used a corrected significance threshold of α = .05/3 = .017.

As with the ratings data, we also conducted omnibus tests of only those sessions with exactly three presentations to validate that unbalanced sessions did not somehow bias the results. These tests (Friedman ANOVAs) revealed significant effects for organization, exact *p* = .0005, engagement, exact *p* = .04, and effectiveness, exact *p* = .003; we found only a marginally significant effect for persuasion, exact *p* = .08. Post-hoc tests (Fisher’s LSD) showed that the audience ranked Prezi presentations higher than PowerPoint and oral presentations on all dimensions, *p*s < .05; PowerPoint and oral presentations were not ranked differently on engagement, persuasion, or effectiveness, *p*s>.05, but the audience did rank PowerPoint presentations as more organized than oral presentations, *p* = .04.

#### Audience omnibus judgments of effectiveness

Before and after the experimental session, audience participants judged the general effectiveness of the three presentation formats. In the pre-survey, they rated each format on its effectiveness for them as presenters and audience members. In the post-survey, they rank-ordered the formats on their “general effectiveness” and were instructed to ignore “how well individual presenters (including today's) use that format.” Although the pre- and post-questions differed in their phrasing and response formats, they nonetheless afford us an opportunity to investigate if and how their judgments changed over the course of the experiment.

**Fig 3:**
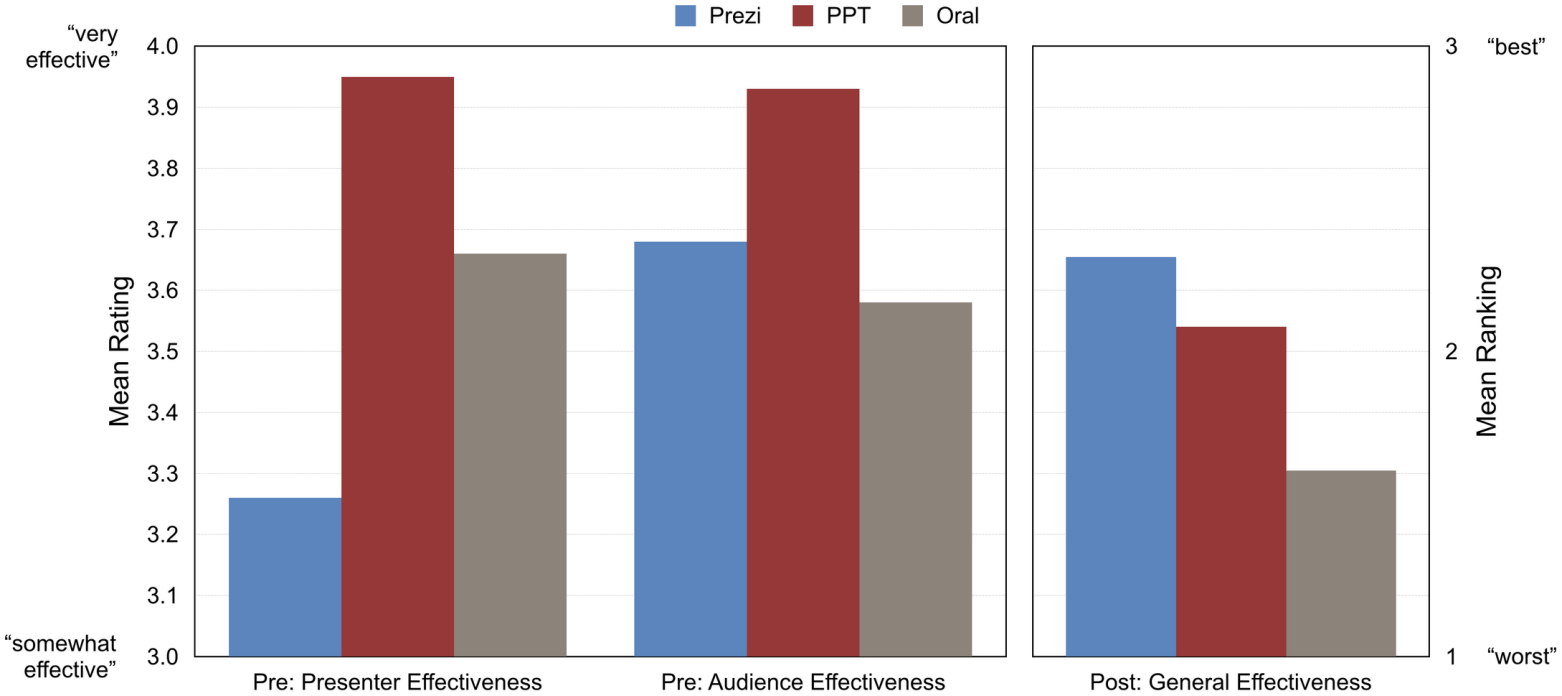
Experiment 1 audience omnibus judgments of effectiveness. Note: Means shown from pre-survey items are calculated based on responses from all participants (as opposed to only those who had experience with all presentation formats).

As already described (see Table 3), the audience began the experiment judging PowerPoint presentations as most effective for presenters and audiences. They ended the experiment, however, with different judgments of efficacy: A majority (52%) ranked Prezi presentations as the most effective, a majority (57%) ranked oral presentations as least effective, and a plurality (49%) ranked PowerPoint presentations second in effectiveness. A Friedman’s ANOVA test (on the mean rankings) confirmed that participants rated presentation formats differently, exact *p* = .00007. Post hoc analysis with Wilcoxon signed-rank tests revealed that the audience ranked both Prezi and PowerPoint presentations as more effective than oral presentations, *ps*≤.003). They did not rank Prezi and PowerPoint presentations significantly differently (*p* = .15). Fig 3 presents these results.

In the pre-survey, some audience participants reported prior experience viewing Prezi presentations but others did not (i.e., those who selected the “not applicable” response option). Compared to participants with no prior experience watching Prezi presentations (*n* = 34), participants with prior Prezi experience (*n* = 117) rated PowerPoint presentations (but not oral presentations) as less effective, *t*(149) = 2.7, *p* = .007, mean difference = .47, and less enjoyable for them, *t*(149) = 2.9, *p* = .004, mean difference = .53. Thus, prior experience with Prezi was associated with negative pre-existing judgments of PowerPoint.

#### Audience correlates of presentation ratings and rankings

What, if any, individual-level variables—demographics and baseline survey responses—correlated with the audience’s judgments of the presentations? If, for example, the more experience the audience had with Prezi, the worse they evaluated those presentations, such a correlation would suggest that the current findings reflect a novelty effect.

We did not find any significant relationships between the audiences’ prior experience with a given presentation format (presenter experience rating, number of years, number of presentations watched last year or lifetime) and their ratings or rank-orderings of that presentation format on any dimensions, all |*r|*s < .16. The only pre-existing audience beliefs about the presentation formats (presenter effectiveness, presenter difficulty, audience effectiveness, audience enjoyableness) that correlated with their ratings or rankings were for oral presentations: the more effective participants rated oral presentations for them as audience members before the experiment, the more effective they rated and ranked oral presentations in the experiment as engaging, *r* = .22 and .26, respectively, *p*s < .01.

Among demographic variables, only age showed reliable correlations with the audiences’ evaluations of presentations: the older the participant, the more effective they rated PowerPoint presentations, *r* = .23, *p* = .007, the more persuasive they ranked PowerPoint presentations, *r* = .24, *p* = .006, and the less organized and persuasive they rated oral presentations, *r* = -.32, *p* = .001, and *r* = -.21, *p* = .01, respectively.

Audience participants’ success in distinguishing better from worse presentations of each format (i.e., their rank-ordering of short expert-created examples) did not correlate with their evaluations of the experimental presentations, nor did it correlate with the audiences’ self-reported experience with each format.

#### Audience free response

Although we cannot assume that participants understood the reasons behind their rank-orderings (cf. [86]), their explanations may nonetheless offer some insight into how they perceived different presentation formats. In explaining their rank-ordering of the presentation formats in terms of their general effectiveness, 8% of participants who preferred Prezi mentioned that it was *new* or *different* or that PowerPoint presentations were *old* or *outdated*. More commonly, they described Prezi as more *engaging* or *interactive* (49%), *organized* (18%), *visually interesting*, *visually compelling*, *visually pleasing*, *sleek*, or *vivid* (15%), or *creative* (13%). Of participants who preferred PowerPoint, 38% described it as more *concise*, *clear*, *easy to follow*, *familiar*, *professional*, or *organized* than the other presentation formats. An equal percentage explained their choice in terms of negative judgments of Prezi, including comments that Prezi was *disorienting*, *busy*, *crowded*, *amateurish*, or *overwhelming*. Participants who rank-ordered oral presentations as most effective remarked that they felt more *engaged* or *connected* with the presenter, could better give their *undivided attention* to the presentation (29%), valued the *eye contact* or *face-to-face interaction* with the presenter (14%), or found presentation software *distracting* (14%).

#### Presenter outcomes and correlates of success

A series of one-way ANOVAs revealed that presentation format did not affect the presenters’ judgments about the business scenario (e.g., “What do you think [Company X] should do?”), self-reported comprehension of the business scenario (“How much do you think you understand the situation with [Company X] and i-Mart?”), or ratings of their own motivation (e.g., “This activity was fun to do”), self-efficacy (e.g., “I think I am pretty good at this activity”), effort (e.g., “I tried very hard on this activity), and effectiveness as presenters (“How convincing do you think your presentation will be to [Company X]’s board members?”); participants using different presentation formats also did not differ in their performance on the multiple-choice test about the business scenario, all *p*s >.05.

The presenter groups did differ in how inclined they were to recommend their presentation format to others (“How likely are you to recommend the presentation tool or presentation format you used to others to make professional presentations?”), *F*(2,144) = 4.2, *p* = .02, with presenters who used Prezi or PowerPoint being more likely to recommend their format than those who made oral presentations, LSD *p* = .03 and *p* = .007, respectively.

Presenter variables—including demographic characteristics and experience with their assigned format—generally did not predict their presentation success, either in terms of audience ratings or rankings. The one exception was that Prezi presenters who were better able to identify effective Prezi presentations were rated and ranked as giving more effective and engaging presentations, .008 < *p*s < .04.

### Discussion

Participants who were randomly assigned to present using Prezi were judged as giving more effective, organized, engaging, and persuasive presentations than those who were randomly assigned to present orally or with PowerPoint. This was true despite the fact that both audience and presenter participants were initially predisposed against Prezi. What might explain these findings?

One explanation is a novelty effect: Perhaps the audience preferred Prezi simply because it is relatively new to them. It appears that this was not the case, however: Only 8% of participants claimed that they preferred Prezi because it was new or different, and there was no significant relationship between the audiences’ experience with Prezi and their ratings or rank-orderings.

Another explanation for these results is that the presenters or audience members were somehow biased towards the Prezi presentations. Again, however, this appears not to be the case. The presenters were least experienced in Prezi, judged themselves least effective presenting with Prezi, and found Prezi presentations hardest to create. We recruited only a small minority (8%) of presenters based on their prior association with Prezi, and used the most conservative exclusion criteria feasible: only individuals without any experience with Prezi or PowerPoint were excluded from participating. All presenters were randomly assigned to their presentation format and were blind to the experimental manipulation. In recruiting audience participants, we did not mention Prezi or PowerPoint, and selected participants only based on their access to Skype and a sufficiently large computer screen. In addition, we minimized contact between the investigator and research participants, and presentations were never identified based on their format; at the end of the experiment, in fact, some participants did not even realize that they had seen a Prezi presentation (as evidenced by their free responses). Data were collected through standardized, online surveys, the investigator was not in the room with the presenter during his or her presentation, and the investigator interacted with the audience only briefly to set up their Skype session. Finally, an analysis of ratings from only the first presentations yielded the same results as the full analysis, making implausible an interpretation based on audience demand characteristics.

Thus, the most likely explanation is that individuals do, in fact, perceive Prezi presentations more favorably than PowerPoint or oral presentation. Experiment 1 has several limitations, however. First, because each audience participant in Experiment 1 was exposed to multiple presentations, we were unable to evaluate presentations on their ultimate goal: to convince the audience (role-playing Company X board members) to accept i-Mart’s business offer. In other words, Experiment 1 demonstrated that Prezi presentations are more effective than other formats in terms of audience perceptions but not decision-making outcomes. Second, we asked the audience about their pre-existing beliefs and prior experiences with PowerPoint, Prezi, and oral presentations at the beginning of the Experiment 1; although it is difficult to imagine how this questioning could have produced the obtained results—particularly given the nature of their pre-existing beliefs and prior experiments—it is a remote possibility. Third, just like the results from any single experiment, the findings of Experiment 1 should be treated cautiously until replicated. We designed a second experiment to address these limitations and extend the findings from the first experiment.

## Experiment 2

In Experiment 2 we showed online participants a single presentation from Experiment 1, and varied randomly which type of presentation (Prezi, PowerPoint, or oral) they viewed. We also randomly assigned some participants to view a presentation on material that was not related to the case material; this control condition served as a baseline that allowed us to estimate the impact of each presentation format. To minimize demand characteristics, we asked participants about their experiences with different presentation formats at the conclusion of the experiment (instead of the beginning), and did not expose participants to multiple presentation formats. Finally, to investigate better the nature of participants’ perceptions about presentation effectiveness, we distinguished between perceptions about the presentation, the presenter, and the audiovisual component of the presentation.

### Methods

#### Participants

We recruited native-English speaking participants via Amazon’s Mechanical Turk using the following language: “In this study, you will read a business case, watch presentations, assume a role, and make a decision.” They were compensated $4 for approximately one hour of their time. Excluding pilot participants who offered us initial feedback on the survey and protocol, 1398 individuals consented to and began the experiment. Of these, 16 participants were excluded because of evidence that they didn’t complete the task properly (e.g., answering a long series of questions identically, incorrectly answering a “trap” question), and 305 were excluded because they dropped out before completing all of the outcome measures, leaving 1069 participants in the final dataset: 272 in the Prezi group, 261 in the PowerPoint group, 275 in the oral presentation group, and 261 in the control group. The number of excluded participants did not covary with group assignment or demographic variables. Table 6 presents demographic information on the included participants.

**Table 6:** Participant demographics for Experiment 2.

Gender identity	Female	557
	Male	512
Mean age (in years)		35.4
SD age (in years)		11.4
Occupation	Student	140
	Other	929
Education level	High school	125
	College (some credit)	393
	College (completed)	413
	Graduate or professional school	138

Note: Unless otherwise stated, numbers refer to number of participants in each category.

#### Stimuli

The main stimuli for this experiment consisted of recorded presentations from Experiment 1. For Prezi and PowerPoint presentations, these were split-screen videos showing the presenter on one side of the screen and the visuals on the other side. For the oral presentations, these were simply audiovisual recordings of the presenter.

Of the 146 presenter participants from Experiment 1, 33 either did not consent to being video-recorded or were not recorded due to technical difficulties. We therefore had a pool of 113 presentation videos to use for Experiment 2: 41 from the Prezi condition (out of a possible 50), 40 from the PowerPoint condition (out of possible 49), and 32 from the oral presentation condition (out of a possible 47). The proportion of presentations that were video-recorded did not vary with their format, exact *p* = .61.

Some of the recorded presentations from Experiment 1 were unusable because of intractable quality issues (e.g., inaudible speech, incomplete video, partially occluded presenter), leaving a total of 89 usable videos (34 Prezi, 28 PowerPoint, 27 oral). The proportion of videos removed because of quality issues did not vary with presentation format, exact *p* = .57.

We randomly selected 25 videos in each format, resulting in a total pool of 75 videos. Because of a URL typo that was not detected until after testing, one PowerPoint video was not presented and participants assigned that video were not able to complete the experiment. Video length varied by format, *F*(2, 71) = 4.2, *p* = .02, with PowerPoint and Prezi presentations lasted longer than oral presentations (*M* = 5.9, 6.0, and 4.6 minutes, respectively).

We were concerned that we could have, perhaps unconsciously, selected better stimuli in the Prezi condition, which would have biased the results. To ensure that our judgments of major audiovisual problems and subsequent exclusion of some videos were not biased, we recruited a separate group of participants to rate the audiovisual quality of the 113 presentation videos. Using the following language, we recruited 455 individuals from Amazon’s Mechanical Turk to serve as judges:

In this study you will judge the technical quality of three short videos. To participate you must have a high-speed Internet connection. We will compensate you $2 for 15–20 minutes of your time.

These participants were totally blind to the experimental hypotheses and manipulation. They completed the audiovisual rating task completely online via the Qualtrics survey platform, and were given the following instructions:

We need your help in determining the audiovisual quality of some Skype presentations we recorded. We want to know which presentations we can use for additional research, and which need to be eliminated due to major technical problems with the recordings. The sorts of technical problems that might exist in some of the videos are: incomplete recordings (the recording starts late or stops early), cropped recordings (the camera isn’t positioned properly), choppy or blurry video, and absent or inaudible audio.

You will watch a single presentation video. Please ignore any aspect of the recording other than its audiovisual quality. In particular, do not base your judgments on the presentation itself, including the presenter’s argument, appearance, or the nature of the accompanying slides. The only thing we care about is whether the audio and video were recorded properly.

Finally, please keep in mind that because these videos were recorded through Skype, even the best recordings are not very high quality.

These judge participants then watched a presentation video (selected at random), rated the quality of its audio and video (on a five-level scale from “very bad” to “very good”), and indicated whether or not there were “any major technical problems with the presentations audio or video”; those who reported major technical problems were asked to identify them.

To address any possibility of experimenter bias—which seemed unlikely, given that we designed the procedure from the outset to guard against such effects—we conducted a series of Presentation Format (Prezi, PowerPoint, oral) x Quality Judgment (inclusion, exclusion) ANOVAs to test 1) whether audiovisual quality was for any reason confounded with presentation format (i.e., the main effect of Presentation Format), 2) whether the excluded videos were indeed lower quality than the included videos (i.e., the main effect of Quality Judgment), and 3) whether our exclusion of videos was biased based on their format (i.e., the interaction between Presentation Format and Audiovisual Quality). We conducted the ANOVAs on the three measures of audiovisual quality collected from the independent judges: ratings of audio quality, ratings of video quality, and judgments of major audiovisual problems.

The results were straightforward: For all three dependent variables, there were no main effects of Presentation Format, *p*s > .13, but we did find a significant main effect of Quality Judgment (with included videos being judged better quality than excluded videos), all *p*s < .002, and did not find any interaction effects, all *p*s > .31. In other words, presentation format was not confounded with audiovisual quality, our judgments of quality corresponded to those of blind judges, and our exclusion of videos was unrelated to presentation format.

#### Procedure

Participants completed the experiment entirely online through Qualtrics. After providing informed consent, and answering preliminary demographic and background questions (e.g., about their familiarity with business concepts and practices) they were told the following:

In this part of the study, you are going to play the role of a corporate executive for [Company X], an innovative clothing company. Another company, i-Mart, wants to sell [Company X’s] t-shirts in its many retail stores. You must decide whether or not to accept i-Mart's offer.

To help you make your decision, we will first provide you with some background on [Company X] and the i-Mart offer. You will see a series of short videos and text that describe relevant aspects of [Company X’s] origins, business model, practices, culture, and community. Please review this background material carefully.

Participants were then shown a series of brief video and textual descriptions of the fictionalized corporate scenario, including information on Company X’s business model, business processes, community, and culture. This material was an abridged version of what Experiment 1 presenter participants studied, but an expanded version of what Experiment 1 audience participants studied.

After viewing the multimedia case material, the participants were asked to identify what product Company X sells (a “trap” question to exclude non-serious participants) and to rate the background material on how engaging it was, how much they enjoyed it, how much they paid attention to it, and how difficult it was to understand.

Participants randomly assigned to the Prezi, PowerPoint, and Oral Presentation conditions were then told the following:

Now that you know a little bit about the company, you will watch a video presentation from another research participant. Just as you are playing the role of a [Company X] executive, the other participant is playing the role of i-Mart's Chief Marketing Office (CMO). In this presentation, he or she will try to convince you and your fellow [Company X] executives to accept i-Mart's offer.

Because this presentation is from another research participant playing the role of an i-Mart executive--and not an actual i-Mart executive--please disregard the presenter's appearance (clothing, age, etc). And because we did not professionally videorecord the presentation, please also try to disregard the relatively poor quality of the video compared to the videos you just viewed.

The purpose of this research is to understand what makes presentations effective. So please listen carefully and do your best to imagine that this is "real".

Identically to Experiment 1, participants rated the presentation on how organized, engaging, realistic, persuasive, and effective it was on a five-level scale from “not at all” to “extremely.” Using the same scale, these participants also rated the *presenter* on how organized, engaging, persuasive, effective, confident, enthusiastic, knowledgeable, professional, nervous, and boring he or she was.

Participants in the Prezi and PowerPoint groups were asked three additional questions. First, they were asked to rate the visual component of the presentation (i.e., the Prezi or the PowerPoint slides) on how organized, engaging, persuasive, effective, dynamic, visually compelling, distracting, informative, distinctive, and boring it was. Second, they were asked to rate whether the presentation had “not enough”, “too much” or an “about right” amount of text, graphs, images, and animations. And finally, there were asked to comment on the visual component of the presentations, including ways in which it could be improved.

All participants then summarized the presentation in their own words, with a minimum acceptable length of 50 characters. Participants were asked to rate how well they understood the “situation with [Company X] and I-Mart,” and to decide whether [Company X] should accept or reject i-Mart’s offer (on a 6-level scale, with the modifiers “definitely,” “probably,” and “possibly”).

In addition, we asked participants a series of recall and comprehension questions about the case. An example recall question is “According to the background materials and the presentation, approximately how many members does [Company X] have?”, with four possible answers ranging from 500,000 to 1.5 million. An example comprehension question is “According to the background materials, what is the biggest challenge [Company X] is facing?”, with possible answers ranging from “marketing” to “logistics.” These comprehension questions were based on the instructor’s guide to the business case material, and included open-ended questions (“Why do you think [Company X] should accept or reject i-Mart's offer?”). At this point we also asked another trap question (“What is 84 plus 27?”).

Finally, and after answering all questions about the business case and presentation, participants answered background questions about their experience with, knowledge of, and general preference for different presentation formats. They also rank-ordered the mini examples of Prezi, PowerPoint, and oral presentations in terms of their effectiveness. These background questions and tasks were the same as those used in Experiment 1.

Participants in the control condition completed the same protocol, with a few exceptions: First, instead of being shown presentations from Experiment 1, they viewed one of three instructional videos (matched for length with the Experiment 1 presentations). Before they viewed these videos they were told “Before you decide what to do about i-Mart's offer to [Company X], we would like you to watch an unrelated presentation and briefly answer some questions about it.” Second, they did not rate how realistic the presentation was, nor did they rate the visual component on how organized, engaging, persuasive, effective, dynamic, visually compelling, distracting, informative, distinctive, and boring it was. And finally, they did not complete the final set of background questions on the different presentation formats or rank-order the example presentations.

### Results

#### Prior experience and pre-existing beliefs

At the outset, participants rated oral and PowerPoint presentations as equally effective in general, and Prezi presentations as less effective than the other two formats. Just as we found in Experiment 1, participants rated themselves as more experienced and effective in making and oral and PowerPoint presentations compared to Prezi presentations. They also rated oral and PowerPoint presentations as more enjoyable and effective for them than viewing Prezi presentations. When asked how difficult it was to make the different types of presentations, they rated Prezi as more difficult than oral and PowerPoint presentations, and oral presentations as more difficult than PowerPoint ones. In terms of the number of presentations watched in the last year and in their lifetime—as well as the number of years of experience—they reported more experience watching oral compared to PowerPoint presentations, and more experience watching PowerPoint than watching Prezi presentations. The same pattern was true for their reported experience in making presentations, with one exception: They reported making more PowerPoint than oral presentations in their lifetime. Table 7 presents full descriptive and inference statistics for all self-reported measures of prior experience with and preexisting beliefs about Prezi, PowerPoint, and oral presentations. The experimental groups did not differ significantly on any of these variables.

**Table 7:** Prior experience and pre-existing beliefs of Experiment 2 participants.

	Means						
	Prezi	PPT	Oral	*F*	N	*p*	post hoc
General effectiveness (rating)	4.92	5.86	5.93	131	471	<0.00001	PPT = Oral > Prezi
Presenter experience (rating)	1.47	3.10	3.14	1030	806	<0.00001	PPT = Oral > Prezi
Presenter effectiveness (rating)	2.93	3.69	3.80	74	297	<0.00001	PPT = Oral > Prezi
Audience effectiveness (rating)	3.42	3.91	3.97	40.7	357	<0.00001	PPT = Oral > Prezi
Audience enjoyableness (rating)	3.26	3.61	3.63	17.6	352	<0.00001	PPT = Oral > Prezi
Presenter difficulty (rating)	4.45	3.39	3.72	68.2	434	<0.00001	Prezi > Oral > PPT
Presentations made last year (#)	0.36	3.81	4.74	37.8	806	<0.00001	Oral > PPT > Prezi
Presentations watched last year (#)	1.14	10.2	12.7	114	804	<0.00001	Oral > PPT > Prezi
Presentations made in lifetime (#)	1.28	68.9	61.8	10.6	798	0.001	PPT > Oral > Prezi
Presentations watched in lifetime (#)	6.1	131	230	41.2	803	<0.00001	Oral > PPT > Prezi
Presenter experience (years)	.56	8.3	12.4	609	804	<0.00001	Oral > PPT > Prezi
Audience experience (years)	1.37	11.8	18.2	443	804	<0.00001	Oral > PPT > Prezi

Note: *F*-statistics and *p*-values are from one-way ANOVAs with Presentation Format as the within-participant factor; for items with significant non-sphericity, we adjusted the *df*s as necessary and confirmed the results with non-parametric Friedman tests. For rating items in which participants could indicate “Not applicable (I have never seen/given this type of presentation),” the reported means, *F*-statistics, sample sizes, and *p*-values include only data from participants who did not select this response option. For rating items, post-hoc inferences are drawn from Fisher’s LSD tests (using all available data); for frequency items, post-hoc inferences are drawn from non-parametric Wilcoxon signed-rank tests due the skewed distributions and presence of outliers.

Most participants (78%) were either “not at all familiar” or “slightly familiar” with Company X, and the modal participant reported being “somewhat experienced” with “concepts and practices from the business world, such as strategy, innovation, product development, sales, and marketing.” The groups did not differ significantly on these variables, nor did they differ on demographic variables such as age, gender, or education.

#### Audience ratings

For overall judgments of the presentations, participants rated Prezi as more organized, effective, engaging, and persuasive than PowerPoint and oral presentations, and rated PowerPoint no differently than oral presentations. They also rated Prezi presenters as more organized, knowledgeable, effective, and professional than PowerPoint presenters and oral presenters; Prezi presenters were not rated differently from other presentations on how nervous, boring, enthusiastic, confident, persuasive, or engaging they were, and PowerPoint presenters were rated no differently than oral presenters on all dimensions. In judging the visual components of the Prezi and PowerPoint presentations, the audience rated Prezi presentations as more dynamic, visually compelling, and distinctive than PowerPoint slides, and marginally more effective and persuasive.

**Fig 4:**
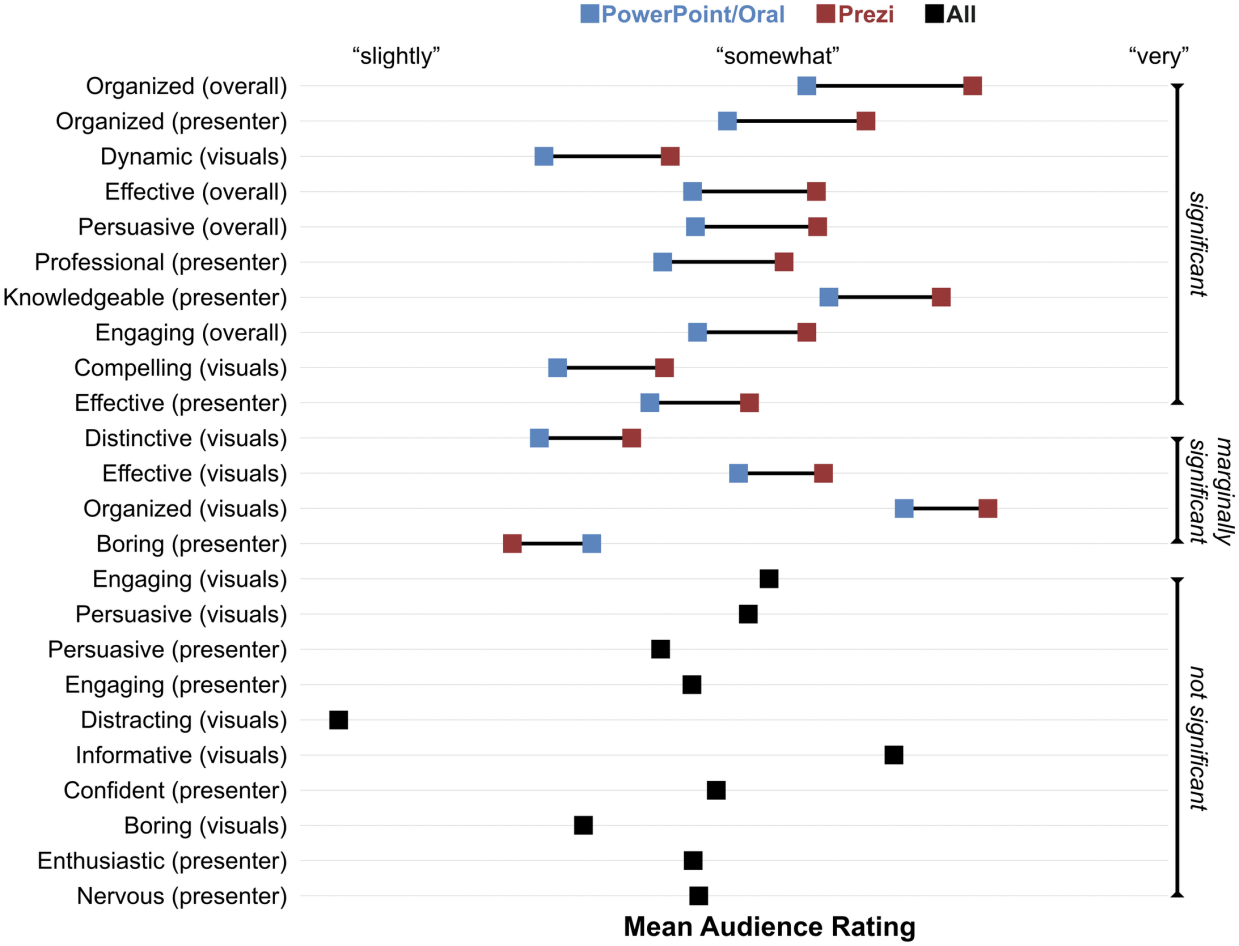
Experiment 2 audience ratings. Note: rating dimensions are ordered by the magnitude of the difference between Prezi and the other presentation formats; for dimensions with no significant differences between presentation formats, only the overall mean is displayed.

Examining the magnitude of mean differences, some effects are clearly larger than others. Most notably, Prezi presentations are rated as most organized and visually dynamic, and Prezi presenters are rated as most organized. Fig 4 and Table 8 present the descriptive and inferential statistics, respectively, for these audience ratings.

**Table 8:** Inferential statistics for Experiment 2 audience ratings.

		Prezi vs PPT			Prezi vs Oral			PPT vs Oral		
		*M*_dif_	*t*	*p*	*M*_dif_	*t*	*p*	*M*_dif_	*t*	*p*
Overall	Organized	0.35	4.3	0.00002	0.52	6.3	<0.00001	0.18	2.1	0.03
	Effective	0.25	2.5	0.01	0.38	4.0	0.00007	0.13	1.3	0.20
	Engaging	0.26	2.6	0.008	0.30	3.1	0.002	0.03	0.3	0.73
	Persuasive	0.28	2.9	0.004	0.34	3.6	0.0003	0.05	0.5	0.59
Presenter	Organized	0.37	4.0	0.00006	0.37	4.1	0.00004	0.01	0.2	0.87
	Effective	0.24	2.4	0.02	0.27	2.8	0.006	0.03	0.3	0.79
	Engaging	0.21	2.0	0.04	0.14	1.4	0.17	-0.07	-0.7	0.45
	Persuasive	0.21	2.1	0.04	0.17	1.8	0.08	-0.05	-0.5	0.65
	Confident	0.18	1.7	0.08	0.09	0.9	0.37	-0.09	-0.9	0.38
	Enthusiastic	0.11	1.1	0.28	0.12	1.2	0.23	-0.01	-0.1	0.95
	Knowledgeable	0.30	3.4	0.0008	0.28	3.2	0.001	-0.02	-0.2	0.83
	Professional	0.25	2.5	0.01	0.37	3.7	0.0002	0.12	1.2	0.25
	Nervous	-0.04	-0.4	0.71	-0.02	-0.2	0.81	0.02	0.2	0.86
	Boring	-0.20	-1.8	0.07	-0.18	-1.7	0.09	0.05	0.5	0.63
Visual	Organized	0.22	2.5	0.01						
	Effective	0.22	2.3	0.02						
	Engaging	0.20	2.1	0.04						
	Persuasive	0.20	2.1	0.03						
	Dynamic	0.33	3.2	0.001						
	Compelling	0.28	2.7	0.006						
	Distracting	-0.17	-1.8	0.08						
	Informative	0.15	1.7	0.09						
	Distinctive	0.24	2.4	0.02						
	Boring	-0.13	-1.2	0.23						

Note: The Prezi vs PPT, Prezi vs oral, and PPT vs oral *t*-tests have 526, 545, and 534 degrees of freedom, respectively. The *p*-values are uncorrected for multiple comparisons; instead we used a corrected significance threshold of α = .05/3 = .017.

The modal participant rated the background case material on Company X as “very engaging” and “completely enjoyable,” reported “mostly” understanding the situation with i-Mart and Company X, and rated the presentations as “very realistic.” Seventy percent of participants expected to do “somewhat well” or “very well” when quizzed about the case. There were no significant group differences on any of these variables.

#### Audience decision-making

Did the presentations actually influence participants’ core judgment of the business scenario and, if so, was one presentation format more effective than others?

**Fig 5:**
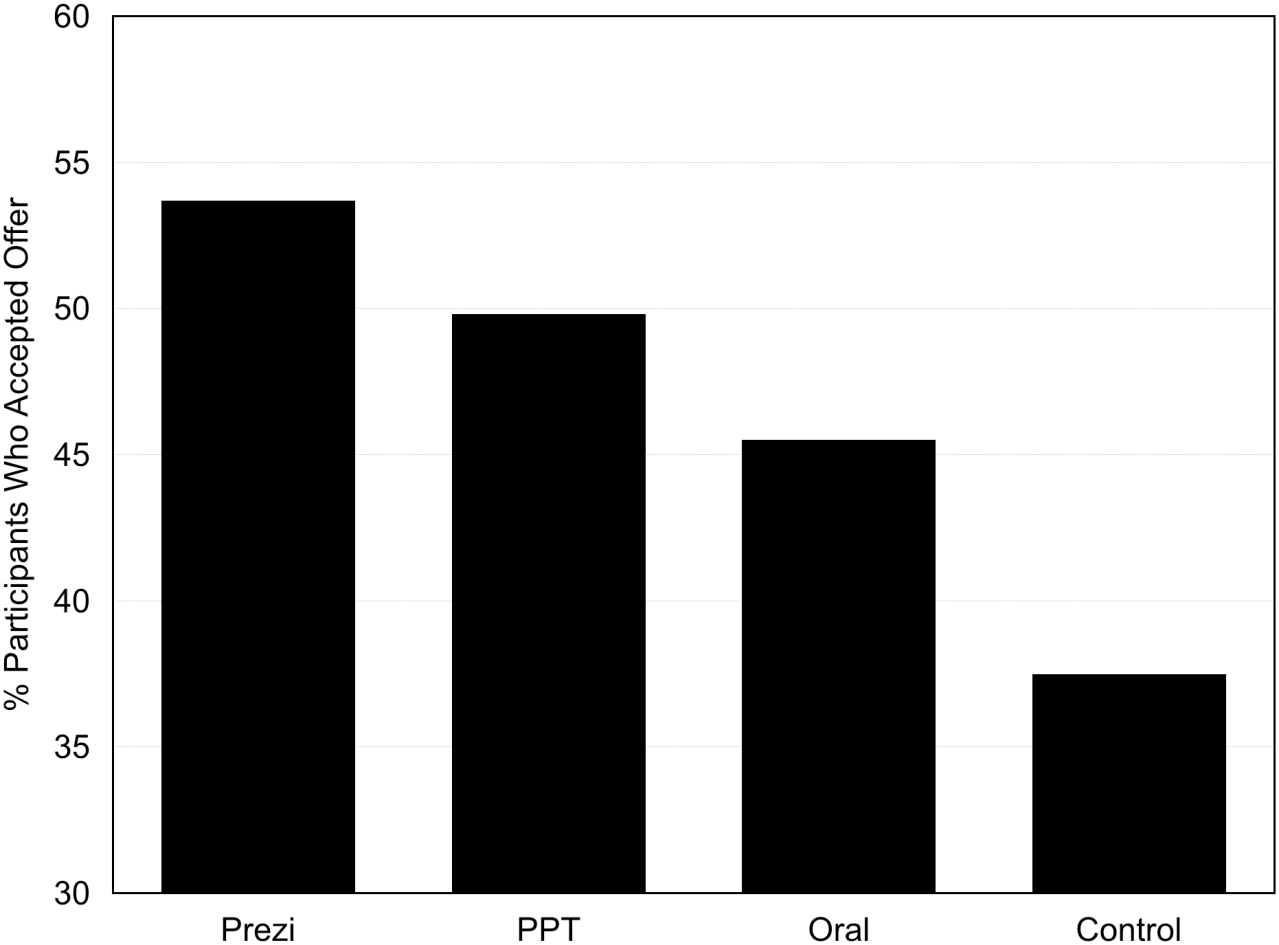
Experiment 2 decision outcomes for each presentation group.

Participants who received a Prezi presentation accepted i-Mart’s offer 53.7% of the time, participants who received a PowerPoint presentation accepted the offer 49.8% of the time, participants exposed to an oral presentation accepted it 45.5% of the time, and participants exposed to the control presentation accepted it 37.5% of the time (see Fig 5). In an omnibus test, these differences were significant, exact *p* = .002. Specific comparisons revealed that Prezi presentations were significantly more influential than control presentations, exact *p* = 0003, marginally more influential than oral presentations, exact *p* = .06, and no more influential than PowerPoint presentations, exact *p* = .39; PowerPoint presentations were significantly more influential than control presentations, exact *p* = .006, but not oral presentations, exact *p* = .34; oral presentations were marginally more influential than control presentations, exact *p* = .07. In order to investigate the impact of presentation software on decision-making, we contrasted the Prezi and PowerPoint groups with the oral presentation groups. We found a marginally significant effect, exact *p* = .06.

On the whole, therefore, the participants’ decision-making results were concordant descriptively (if not always inferentially) with the rating results.

If participants’ perceptions of the presentations and decisions about the case were both influenced by presentation format, then we would expect them to be associated with each other. And this is indeed what we found. Excluding participants in the control group (who did not make judgments about comparable presentations), those who rejected the i-Mart offer rated presentations as worse than those who accepted the i-Mart offer. This was true for 23 of the 24 rating dimensions (“visually boring” was the exception), with the largest effects for ratings of effectiveness and persuasiveness. Those who rejected the offer rated the overall presentation, visual aids, and presenter as less effective than those who accepted the offer, with effect sizes (Cohen’s *d*) of .93, .83, and .78, respectively. These effects were consistent across formats, all interaction *p*s > .05.

We conducted an analogous set of analyses that preserved the original 6-level scale of the decision variable (“possibly accept,” “probably accept,” “definitely accept,” “possibly reject,” “probably reject,” “definitely reject”). These analyses produced qualitatively identical results, both in terms of decision-making as a function of group assignment and the correlation between decision-making and presentation ratings.

#### Memory and comprehension

Participants’ performance on the four rote memory questions did not vary across conditions, nor did their correct identification (according to the case designers) of reasons to accept or reject the offer, with one exception: Compared to those in the treatment groups, control participants were more likely to identify Company X’s ability to meet production demand as a reason to reject the i-Mart, omnibus exact *p* = .00004.

#### Correlates of presentation outcomes

There were no notable correlations between demographic variables and participants’ ratings or decisions. In particular, participants’ experience with or preexisting beliefs about each presentation format did not correlate with their ratings of the experimental presentations, mirroring the results from Experiment 1 (but with much greater statistical power). Presentation length or recording quality (as assessed by the independent judges) did not correlate with presentation outcomes.

Participants’ success in distinguishing better from worse presentations of each format—that is, their rank-ordering of short expert-created examples—correlated slightly with their evaluations of the presentations. Most notably, the better participants did on the rank-ordering PowerPoint task, the worse they rated PowerPoint (but not Prezi) presentations on visual dimensions; the same was true for the Prezi task and presentations. For example, participants’ performance in the PowerPoint task correlated negatively with their judgments of how “visually dynamic” PowerPoint presentations were, *r* = -.22, *p* = .0005, and participants’ performance on the Prezi task correlated negatively with their judgments of how “visually dynamic” Prezi presentations were, *r* = -.16, *p* = .009. Thus, individuals with more expertise in PowerPoint and Prezi were more critical of PowerPoint and Prezi presentations, respectively.

#### Audiovisual attributes of Prezi and PowerPoint presentations

To understand the media attributes and psychological mechanisms that underlie the observed effects of format, we examined how participants’ judgments about amount of text, graphs, animations, and images in the presentations correlated with their judgments of the presentations, the visual component of the presentations, and the presenters themselves. To examine these relationships, we conducted one-way ANOVAs with the various ratings as the dependent variables, and participants’ judgments (“not enough,” “about right,” “too much”) about the amount of text, graphs, animations, and images in the PowerPoint and Prezi presentations as the independent variable. For nearly all (80 of 96) of these ANOVAs, the results were highly significant, *p*s < .001. In judging the amount of text, participants typically rated “too much” or “not enough” text as worse than an “about right” amount; in judging graphs, images, and animations, participants typically rated “too much” and “just right” both as equally better than “not enough.” Averaging across all rating dimensions, the text and graph effects were over twice as large as the animation and image effects; averaging across all attributes, the effects for visual ratings was over twice as large as the effects for presenter and overall ratings. Participants’ judgments about the media attributes of presentations did, therefore, relate to their overall assessments of the presenters and presentations.

Summing across PowerPoint and Prezi presentations, the modal participant indicated that there was the “about right” amount of text, graphs, animations, and images. Only 21% of participants thought there was not enough or too much text; for the other dimensions, this percentage ranged from 42–51%. More participants indicated that there was not enough text, graphs, and animations in PowerPoint presentations than Prezi presentations, with animation as the most distinguishing attribute. Table 9 presents the descriptive and inferential statistics for these variables.

**Table 9:** Experiment 2 judgments of audiovisual attributes by presentation format.

	“not enough”	“about right”	“too much”
	Text (exact *p* = .02)		
Prezi	25	225	22
PPT	45	190	21
	Graph (exact *p* = 0.05)		
Prezi	98	158	16
PPT	110	140	6
	Animation (exact *p* = 0.001)		
Prezi	105	145	22
PPT	134	114	8
	Images (exact *p* = 0.32)		
Prezi	96	165	11
PPT	101	140	15

As shown in Table 10, participants’ judgments about the audiovisual attributes of the Prezi and PowerPoint presentations were associated with the decision about the business scenario. Individuals who reported that there was not enough text, graph, animation, or images tended to reject the offer for i-Mart, whereas those who reported that there was the “about right” amount of those attributes tended to accept the offer. This effect was particularly pronounced for judgments of graphs and text. Participants who reported too much text also tended to reject the offer.

**Table 10:** Experiment 2 judgments of audiovisual attributes by decision outcome.

	“not enough”	“about right”	“too much”
	Text (exact *p* = .0003)		
reject	45	180	28
accept	25	235	15
	Graph (exact *p* = 0.00003)		
reject	125	119	9
accept	83	179	13
	Animation (exact *p* = 0.10)		
reject	126	112	15
accept	113	147	15
	Images (exact *p* = 0.04)		
reject	108	135	10
accept	89	170	16

In sum, participants’ perceptions of presenters and the presentations correlated with their evaluations of the amount of text, graphs, images, and animations that were included in the presentations. Presenters and presentations were rated worse if they had too much or not enough text, and not enough graphs, images, and animations; in terms of audience decision-making, presentations were less effective if they contained too much or not enough text, or not enough graphs, animations, and images. PowerPoint presentations were judged to have too little of all attributes, particularly animation.

### Discussion

Replicating results from Experiment 1, participants rated presentations made with Prezi as more organized, engaging, persuasive, and effective than both PowerPoint and oral presentations. This remained true despite participants’ preexisting bias against Prezi and the different context of Experiment 2: the audience did not view multiple presentations of different formats and presentations were prerecorded instead of live. Extending the Experiment 1 results, participants also judged Prezi presentations as better in various ways (e.g., more visually compelling, more dynamic) than PowerPoint presentations; participants even rated Prezi presenters more highly (e.g., more knowledgeable, more professional) than PowerPoint presenters.

In making decisions as corporate executives, participants were persuaded by the presentations. Compared to the baseline decisions of the control group, those in the treatment group shifted their decisions by 16.2%, 12.3%, and 8.0% depending on whether they viewed Prezi, PowerPoint, or oral presentations, respectively. The non- or marginal significance of some between-format comparisons (e.g., PowerPoint versus Prezi) is difficult to interpret. We hesitate to dismiss these differences as statistical noise given their general alignment with rating results, as well as the correlation between business decisions and presentation ratings (which do vary significantly with format). For the more objective outcome of decision-making, we can, at the very least, provisionally conclude that Prezi presentations are more effective than oral presentations, and that software-aided presentations are more effective than oral presentations.

We did not find any evidence that the presentations affected participants’ memory or understanding of the case, nor did we find evidence that certain presentation formats impacted learning more than others. Given the goals of the presentations and design of the experiment, however, we hesitate to draw any conclusions from these null results.

## General discussion

The most important finding across the two experiments is easy to summarize: Participants evaluated Prezi presentations as more organized, engaging, persuasive, and effective than both PowerPoint and oral presentations. This finding was true for both live and prerecorded presentations, when participants rated or ranked presentations, and when participants judged multiple presentations of different formats or only one presentation in isolation. Results from Experiment 2 demonstrate that these presentations influenced participants’ core judgments about a business decision, and suggest that Prezi may benefit both behavioral and experiential outcomes. We have no evidence, however, that Prezi (or PowerPoint or oral presentations) facilitate learning in either presenters or their audience.

Several uninteresting explanations exist for the observed Prezi effects, none of which posit any specific efficacy of Prezi or ZUIs in general: namely, novelty, bias, and experimenter effects. We consider each in turn.

Novelty heavily influences both attention and memory [87, 88], and the benefits of new media have sometimes dissipated over time—just as one would expect with novelty effects [3]. However, we found no evidence that novelty explains the observed benefits of Prezi: Participants who were less familiar with Prezi did not evaluate Prezi presentations more favorably, and only a small fraction of participants who favored Prezi explained their preference in terms of novelty. We therefore are skeptical that mere novelty can explain the observed effects.

We also considered the possibility that participants had a pre-existing bias for Prezi. This seems unlikely because presenter participants were selected based only on minimal experience with both PowerPoint and Prezi and were assigned randomly to the experimental groups; audience participants from both experiments were selected based merely on high-speed internet access, and the words “Prezi” and “PowerPoint” were not used in any audience recruitment material. In fact, both sets of participants entered the research with biases against Prezi, not for Prezi: They reported more experience with PowerPoint and oral presentations than Prezi, and perceived PowerPoint and oral presentations as more (not less) efficacious than Prezi. Thus, we reject the idea that the results simply reflect pre-existing media biases.

For many reasons, we also find it unlikely that experimenter effects—including demand characteristics (i.e., when participants conform to the experimenters’ expectations)—can explain the observed effects. First, at the outset we did not have strong hypotheses about the benefits of one format over the others. Second, the results are subtle in ways that neither we nor a demand characteristics hypothesis would predict: the effects on subjective experience diverged somewhat from the effects on decision-making, and there were no memory or comprehension effects. Third, the between-participants design of Experiment 2 (and between-participants analysis of Experiment 1) limited participants’ exposure to a single presentation format, thereby minimizing their ability to discern the experimental manipulation or research hypotheses. Fourth, we ensured that the presentations were equally high-quality; we did not unconsciously select Prezi presentations that happened to be higher quality than presentations in the other formats. Fifth, the random assignment of presenters to format limits the possible confounding of presenter variables with presentation formats or qualities; and no confounding with format was observed in presenters’ preexisting beliefs, prior experience, or demographics. And finally, in Experiment 2 we only explicitly mentioned or asked participants questions about Prezi, PowerPoint, and oral presentations at the conclusion of the experiment, after collecting all key outcome data.

We therefore conclude that the observed effects are not confounds or biases, but instead reflect a true and specific benefit of Prezi over PowerPoint or, more generally, ZUIs over slideware. If, however, these experimental effects merely reveal that Prezi is more user-friendly than PowerPoint—or that PowerPoint’s default templates encourage shallow processing by “[fetishizing] the outline at the expense of the content” [89] (pB26)—then we have learned little about the practice or psychology of communication. But if these effects instead reflect intrinsic properties of ZUIs or slideware, then they reveal more interesting and general insights about effective communication.

It is difficult to understand Prezi’s benefits in terms of user-friendliness because the odds were so clearly stacked in PowerPoint’s favor. Presenters were much more experienced in using PowerPoint than Prezi and rated PowerPoint as easier to use than Prezi. Especially given the task constraints—participants only had 45 minutes to prepare for a 5-minute presentation on a relatively new, unfamiliar topic—Prezi’s user interface would have to be improbably superior to PowerPoint’s interface to overcome these handicaps. Moreover, participants’ prior experience with PowerPoint or Prezi did not correlate with their success as presenters, as one would expect under an ease-of-use explanation. Finally, audience participants did not simply favor the Prezi presentations in an even, omnibus sense—they evaluated Prezi as better in particular ways that align with the purported advantages of ZUIs over slideware. This pattern of finding makes most sense if the mechanism were at the level of media, not software.

Participants’ evaluations of Prezi were particularly telling in three ways. First, in participants’ own words (from Experiment 1), they frequently described Prezi as *engaging*, *interactive*, *visually compelling*, *visually pleasing*, or *vivid*, and PowerPoint as *concise*, *clear*, *easy to follow*, *familiar*, *professional*, or *organized*. Second, in participants’ ratings (from Experiment 2), the visuals from Prezi presentations were evaluated as significantly more dynamic, visually compelling, and distinctive than those from PowerPoint presentations. And third, in judging the audiovisual attributes of presentations, participants’ identified animations as both the attribute most lacking in presentations and the attribute that most distinguished Prezi from PowerPoint; furthermore, the more a presentation was judged as lacking animation, the worse it was rated. Taken together, this evidence suggests that Prezi presentations were not just better overall, but were better at engaging visually with their audience through the use of animation. Because ZUIs are defined by their panning and zooming animations—and animation is an ancillary (and frequently misused) feature of slideware—the most parsimonious explanation for the present results is in terms of ZUIs and slideware in general, not Prezi and PowerPoint in particular. The medium is not the message, but it may be the mechanism.

The animated nature of ZUIs makes more sense as possible mechanism for the observed effects when one considers relevant literature on animation. Past research has shown that animation can induce physiological and subjective arousal (e.g., [90, 91]) and facilitate attention, learning, and task performance (e.g., [92–94]; but see also [95, 96]). Most pertinently, people appear to prefer animated media over static media. Participants rate animated online advertisements as more enjoyable, persuasive, effective, and exciting than static online advertisements [97, 98], animated websites as more likeable, engaging, and favorable than static websites [99], and animated architectural displays as clearer than static displays [100]. In an experiment of online academic lectures, participants preferred whiteboard-style animations over a slideware-style version matched for both visual and audio content [101]. Moreover, ZUI’s use of animation aligns with recommended principles for using animation effectively in presentations, which include the creation of a large virtual canvas and the use of zooming to view detail [102]. Slideware, on the other hand, encourages the use of superfluous animation in slide transitions and object entrances/exits, despite evidence that adding such “seductive details” to multimedia presentations can be counterproductive [72].

Therefore, we not only conclude that audiences prefer Prezi over PowerPoint presentations, but also conclude that their preference is rooted in an intrinsic attribute of ZUIs: panning and zooming animations. Compared to slideware’s sequential, linear transitions (and oral presentations’ total lack of visual aids), zooming and panning over a virtual canvas is a more engaging and enjoyable experience for an audience.

From this perspective, the reason that participants rated Prezi presentations as more persuasive, effective, and organized than other presentations—and Prezi presenters as more knowledgeable, professional, effective, and organized than other presenters—was because they confuse media with messages and messengers. Dual-process models of persuasion contend that opinion change occurs through not just slow deliberations grounded in logic and reason but also through fast shortcuts rooted in associations and cues [103–106]. If better presenters with better arguments tend to give better presentations, then an audience’s experience while viewing a presentation may shade their judgments about its presenter or argument. This is the same basic logic of research that demonstrates PowerPoint’s persuasion advantage over oral presentations [53, 54]. Just as audiences appear more persuaded by slideware than by oral presentations, they also appear more persuaded by ZUI than by slideware presentations. But unlike past research, we do not argue that audience members use technological sophistication as a cue for argument quality [53] or presenter preparedness [54]; instead, we suggest that they use their subjective viewing experience as a heuristic for judging both presentations and presenters. Because ZUI presentations are more engaging than slideshows, ZUI presentations and presenters are judged more positively than slideshows.

## Concluding remarks

Media research, including research into presentation software, is plagued methodologically by a lack of experimental control, the unjustifiable assumption that media effects are constant across individuals and content, and a failure to account for the biases of all involved: the presenters, the audiences, and the researchers. In the research reported here we strived to overcome these challenges by randomly assigning presenters and audience members to competing presentation formats, blinding them to the experimental manipulations, and sampling a sufficient array of presentations within each format.

Our conclusions about the advantages of ZUIs (such as Prezi) over slideware (such as PowerPoint) and oral presentations are, of course, tentative. Further research will need to replicate the findings across different presentation contexts, clarify whether the subjective benefits of ZUIs over slideware result in decision-making or behavioral advantages, and better investigate the precise media attributes responsible for these advantages. Like others [107], we caution against technological determinism: Presentation medium is but one of many factors that determine presentation success, and presentations that rely on any given medium can succeed or fail. Because slideware can be used to zoom and pan over a virtual canvas just as ZUIs can be used to create slideshows, the benefits of ZUIs over slideware are ultimately based on affordances: How much do certain formats encourage or enable psychologically advantageous media attributes, such as zooming and panning animations?

In many ways, it is surprising that we found any effects of presentation medium. The presentations differed in many ways aside from their format, ways that surely influenced their effectiveness: Each presentation was made by a different person (sampled from a diverse pool of participants), presenters chose what content to include in their presentation, and presenters decided how to convey that content within their assigned format. Under real-world circumstances in which presentations of different formats are actually contrasted with each other, we expect this background “noise” to be greatly reduced and impact of format correspondingly greater.

## Supporting information

S1 File Experiment 1 audience pre-survey. (PDF)

S2 File Experiment 1 audience post-survey. (PDF)

S3 File Experiment 1 presenter pre-survey. (PDF)

S4 File Experiment 1 presenter post-survey. (PDF)

S5 File Experiment 2 audience post-survey. (PDF)
