# Correction: Does a presentation's medium affect its message? PowerPoint, Prezi, and oral presentations

**DOI:** 10.1371/journal.pone.0186673

**Published:** 2017-10-12

**Authors:** The PLOS ONE Staff

There are errors in the Funding Section. The correct funding information is as follows: This research was supported by a grant from Prezi (http://www.prezi.com) to SMK. In the sponsored research agreement and in our conversations with Prezi leadership, they agreed to let us conduct the study as we wished and publish it no matter what the results revealed. Aside from funding the research, the only role that any employees of Prezi played was (as documented in the manuscript) 1) to provide us with a distribution list of Boston-area Prezi customers (8 of whom participated in the first experiment) and 2) as experts in Prezi, review the background questionnaire to ensure that we were accurately describing Prezi’s purported benefits and features (just as PowerPoint and oral presentation experts did the same). No employees at Prezi had any role in the study design, data collection and analysis, decision to publish, or preparation of the manuscript. None of the authors have any professional or financial connection to Prezi or substantive personal relationships with any Prezi employees. We do not plan to conduct any follow-up research on this topic and will not solicit or accept any future funding from Prezi. As evident in the manuscript, we took special care not to allow bias or demand characteristics to influence this research. The publisher apologizes for the error.
