# Supplementary material for: Does a presentation’s medium affect its message? PowerPoint, Prezi, and oral presentations
Source: PLoS One. 2017 Jul 5;12(7):e0178774. doi: 10.1371/journal.pone.0178774 (PMC5497950; doi:10.1371/journal.pone.0178774)
Supplement: S1 File — (PDF) [file pone.0178774.s001.pdf]

Welcome! Thank you for completing this brief questionnaire, which should take approximately 5 minutes to complete. Your thoughtfulness and honesty in answering these questions is extremely important to us. Finally, it is important that you complete this survey in one sitting, and do not try to answer any of the questions by googling things in another browser window or tab.

ID Please tell us about yourself:

Contact email address:

Age (in years):

Current occupation:

Your initials:

Education What is the highest level of education?

- ❍ No schooling completed (1)
- ❍ Nursery school to 8th grade (2)
- ❍ Some high school, no diploma (3)
- ❍ High school graduate, diploma or the equivalent (for example: GED) (4)
- ❍ Some college credit, no degree (5)
- ❍ Trade/technical/vocational training (6)
- ❍ Associate degree (7)
- ❍ Bachelor’s degree (8)
- ❍ Master’s degree (9)
- ❍ Professional degree (10)
- ❍ Doctorate degree (11)

Language Which of the below best describes you?

- ❍ I am a native English speaker
- ❍ I am not a native English speaker but fluent in English
- ❍ I am not a native English speaker and am not fluent in English

Gender What is your gender identity?

- ❍ Male (1)
- ❍ Female (2)
- ❍ Other (3)

A note on language: When we use the term "presentation", we mean a formal, planned, and oral presentation of any duration, including a public speech, an academic lecture, a webinar, a class presentation, a wedding toast, a sermon, a product demonstration, a business presentation, and so on. Examples of things we do NOT mean are: a theatrical performance, an impromptu toast at dinner, and any presentation with no audience. When we say PowerPoint presentations, we mean presentations that were made using Microsoft PowerPoint, not other software such as Apple's Keynote. When we say Prezi presentations, we mean presentations that were made using Prezi presentation software. Also, when we refer to "oral presentation", we mean a presentation that is only spoken and does not include any visual aids or the use of presentation software.

Experience How experienced are you at making the following types of presentations?

	Not at all Experienced (1)	Slightly Experienced (2)	Somewhat Experienced (3)	Very Experienced (4)	Extremely Experienced (5)
Oral Presentation	❍	❍	❍	❍	❍
PowerPoint	❍	❍	❍	❍	❍
Prezi	❍	❍	❍	❍	❍

Effective_1 When you give a presentation, how effective the following presentations for you?

	Not at all Effective (1)	Slightly Effective (2)	Somewhat Effective (3)	Very Effective (4)	Extremely Effective (5)	N/A (I have never given this type of presentation) (99)
Oral Presentation	❍	❍	❍	❍	❍	❍
PowerPoint	❍	❍	❍	❍	❍	❍
Prezi	❍	❍	❍	❍	❍	❍

Effective_2 When somebody else gives a presentation, how effective the following types of presentations for you?

	Not at all Effective (1)	Slightly Effective (2)	Somewhat Effective (3)	Very Effective (4)	Extremely Effective (5)	N/A (I have never seen this type of presentation) (99)
Oral Presentation	❍	❍	❍	❍	❍	❍
PowerPoint	❍	❍	❍	❍	❍	❍
Prezi	❍	❍	❍	❍	❍	❍

Enjoyable When somebody else gives a presentation, how enjoyable the following types of presentations for you?

	Not at all Enjoyable (1)	Slightly Enjoyable (2)	Somewhat Enjoyable (3)	Very Enjoyable (4)	Extremely Enjoyable (5)	N/A (I have never seen this type of presentation) (99)
Oral Presentation	❍	❍	❍	❍	❍	❍
PowerPoint	❍	❍	❍	❍	❍	❍
Prezi	❍	❍	❍	❍	❍	❍

Ease How easy or difficult is it for you to make the following types of presentations?

	Not at all Difficult (1)	Slightly Difficult (2)	Somewhat Difficult (3)	Very Difficult (4)	Extremely Difficult (5)	N/A (I have never made this type of presentation) (99)
Oral Presentation	❍	❍	❍	❍	❍	❍
PowerPoint	❍	❍	❍	❍	❍	❍
Prezi	❍	❍	❍	❍	❍	❍

Freq_year In the last year, approximately how many of the following types of presentations did watch? (Please write "0" if you have not watched any)

Oral Presentation:
PowerPoint:
Prezi:

Freq_life In your lifetime, approximately how many of the following types of presentations did you watch? (Please write "0" if you have not watched any)

Oral Presentation:

PowerPoint:

Prezi:

Year For approximately how many years have you been watching the following types of presentations?

Oral Presentation:

PowerPoint:

Prezi:

Next we will ask you to rate the quality of very short (10-20 second) samples of Prezi presentations. These videos have no sound or oral narration—this is on purpose so that you evaluate them based only on their visual aspects.

Prezi1 Please watch the four videos below, one at a time. When you are done watching them all, rank them from best to worst by clicking the button on the right side (1=best, 2 = good, 3 = bad, 4=worst). Feel free to watch the videos more than once if that helps you better evaluate them.

	1	2	3	4
Prezi1_video1https://s3.amazonaws.com/prz/Relation_1.mp4				
Prezi1_video2https://s3.amazonaws.com/prz/Relation_2.mp4				
Prezi1_video3https://s3.amazonaws.com/prz/Relation_3.mp4				
Prezi1_video4https://s3.amazonaws.com/prz/Relation_4.mp4				

Prezi2 Please watch the four videos below, one at a time. When you are done watching them all, rank them from best to worst by clicking the button on the right side (1=best, 2 = good, 3 = bad, 4=worst). Feel free to watch the videos more than once if that helps you better evaluate them.

	1	2	3	4
Prezi2_video1https://s3.amazonaws.com/prz/Zoom_1.mp4				
Prezi2_video2https://s3.amazonaws.com/prz/Zoom_2.mp4				
Prezi2_video3https://s3.amazonaws.com/prz/Zoom_3.mp4				
Prezi2_video4https://s3.amazonaws.com/prz/Zoom_4.mp4				

Prezi3 Please watch the four videos below, one at a time. When you are done watching them all, rank them from best to worst by clicking the button on the right side (1=best, 2

= good, 3 = bad, 4=worst). Feel free to watch the videos more than once if that helps you better evaluate them.

	1	2	3	4
Prezi3_video1https://s3.amazonaws.com/prz/metaphor_1.mp4				
Prezi3_video2https://s3.amazonaws.com/prz/metaphor_2.mp4				
Prezi3_video3https://s3.amazonaws.com/prz/metaphor_3.mp4				
Prezi3_video4https://s3.amazonaws.com/prz/metaphor_4.mp4				

thanks Thank you for completing the survey. You are qualified to participate. Please view the available time-slots for this week below: CALENDAR Send your preferred time-slot to [email address]. If there is not a time slot that works for you currently, check the calender next week (In that case save the link to the calendar). The new time slots will be made available one week at a time.

ID Please fill in the form below.
ID:
Initials:

welcome In this activity, you will rate the quality of multiple presentations. A note on language: When we use the term "presentation", we mean a formal, planned, and oral presentation of any duration, including a public speech, an academic lecture, a webinar, a class presentation, a wedding toast, a sermon, a product demonstration, a business presentation, and so on. Examples of things we do NOT mean are: a theatrical performance, an impromptu toast at dinner, and any presentation with no audience. Also, when we refer to "oral presentation", we mean a presentation that is only spoken and does not include any visual aids or the use of presentation software. Some presentations do not have narrative. This is on purpose. We want you to focus on the visual aspects on these presentations. Click next, when you are ready.

PPT1 Please watch the four videos below, one at a time. When you are done watching them all, rank them from best to worst by clicking the button on the right side (1=best, 2=good, 3=bad, 4=worst). Feel free to watch the videos more than once if that helps you better evaluate them.

	1	2	3	4
PPT1_video1https://s3.amazonaws.com/prz/Graphic_1.mp4				
PPT1_video2https://s3.amazonaws.com/prz/Graphic_2.mp4				
PPT1_video3https://s3.amazonaws.com/prz/Graphic_3.mp4				
PPT1_video4https://s3.amazonaws.com/prz/Graphic_4.mp4				

PPT2 Please watch the four videos below, one at a time. When you are done watching them all, rank them from best to worst by clicking the button on the right side (1=best, 2=good, 3=bad, 4=worst). Feel free to watch the videos more than once if that helps you better evaluate them.

	1	2	3	4
PPT2_video1https://s3.amazonaws.com/prz/charts_1.mp4				
PPT2_video2https://s3.amazonaws.com/prz/charts_2.mp4				
PPT2_video3https://s3.amazonaws.com/prz/charts_3.mp4				
PPT2_video4https://s3.amazonaws.com/prz/charts_4.mp4				

PPT3 Please watch the four videos below, one at a time. When you are done watching them all, rank them from best to worst by clicking the button on the right side (1=best, 2=good, 3=bad, 4=worst). Feel free to watch the videos more than once if that helps you better evaluate them.

	1	2	3	4
PPT3_video1 https://www.youtube.com/watch?v=T9NntA2Ny4A				
PPT3_video2 https://www.youtube.com/watch?v=tNL2nZ4nZ7c				
PPT3_video3 https://www.youtube.com/watch?v=atQSf4ON6Xs				
PPT3_video4 https://www.youtube.com/watch?v=liXffWyMhxk				

Speaking1 Please watch the four videos below, one at a time. When you are done watching them all, rank them from best to worst by clicking the button on the right side (1=best, 2=good, 3=bad, 4=worst). Feel free to watch the videos more than once if that helps you better evaluate them.

	1	2	3	4
Speaking1_video1 [video available by request]				
Speaking1_video2 [video available by request]				
Speaking1_video3 [video available by request]				
Speaking1_video4 [video available by request]				

Speaking2 Please watch the four videos below, one at a time. When you are done watching them all, rank them from best to worst by clicking the button on the right side (1=best, 2=good, 3=bad, 4=worst). Feel free to watch the videos more than once if that helps you better evaluate them.

	1	2	3	4
Speaking2_video1<br>[video available by request]				
Speaking2_video2<br>[video available by request]				
Speaking2_video3<br>[video available by request]				
Speaking2_video4<br>[video available by request]				

Speaking3 Please watch the four videos below, one at a time. When you are done watching them all, rank them from best to worst by clicking the button on the right side (1=best, 2=good, 3=bad, 4=worst). Feel free to watch the videos more than once if that helps you better evaluate them.

	1	2	3	4
Speaking3_video1<br>[video available by request]				
Speaking3_video2<br>[video available by request]				
Speaking3_video3<br>[video available by request]				
Speaking3_video4<br>[video available by request]				
