# Supplementary material for: Does a presentation’s medium affect its message? PowerPoint, Prezi, and oral presentations
Source: PLoS One. 2017 Jul 5;12(7):e0178774. doi: 10.1371/journal.pone.0178774 (PMC5497950; doi:10.1371/journal.pone.0178774)
Supplement: S2 File — (PDF) [file pone.0178774.s002.pdf]

IDs Please enter the information below:
Your ID number:
Your initials:
1st Presenter's ID number:

Please watch the first presentation now and click next when the presentation is over. Thank you!

Evaluate Overall, how ______ was this presentation?

	Not at all (1)	Slightly (2)	Somewhat (3)	Very (4)	Extremely (5)
Organized	❍	❍	❍	❍	❍
Engaging	❍	❍	❍	❍	❍
Realistic	❍	❍	❍	❍	❍
Persuasive	❍	❍	❍	❍	❍
Effective	❍	❍	❍	❍	❍

Comment1 Do you have any comments about the presentation and the presenter? How do you think it can be improved?

Next, you will be listening Presentation #2. When the presentation is over, click next.

ID_Pres2 Please enter the information below:
2nd Presenter's ID number:

Eval_Pres2 Overall, how ______ was this presentation?

	Not at all (1)	Slightly (2)	Somewhat (3)	Very (4)	Extremely (5)
Organized	❍	❍	❍	❍	❍
Engaging	❍	❍	❍	❍	❍
Realistic	❍	❍	❍	❍	❍
Persuasive	❍	❍	❍	❍	❍
Effective	❍	❍	❍	❍	❍

Comment2 Do you have any comments about the presentation and the presenter? How do you think it can be improved?

Next, you will be listening Presentation #3. When the presentation is over, click next.

ID_Pres3 Please enter the information below:
3rd Presenter's ID number:

Eval_Pres3 Overall, how ______ was this presentation?

	Not at all (1)	Slightly (2)	Somewhat (3)	Very (4)	Extremely (5)
Organized	❍	❍	❍	❍	❍
Engaging	❍	❍	❍	❍	❍
Realistic	❍	❍	❍	❍	❍
Persuasive	❍	❍	❍	❍	❍
Effective	❍	❍	❍	❍	❍

Comment3 Do you have any comments about the presentation and the presenter? How do you think it can be improved?

Next, you will compare the presentations on four different dimensions and on their overall effectiveness.

Comp_Org Please rank the below presentations based on how organized they were? (1 - the best, 2 - intermediate, 3 - the worst)
______ 1st presentation
______ 2nd presentation
______ 3rd presentation

Rank_engagine Please rank the below presentations based on how engaging they were? (1 - the best, 2 - intermediate, 3 - the worst)
______ 1st presentation
______ 2nd presentation
______ 3rd presentation

Rank_persuasive Please rank the below presentations based on how persuasive they were? (1 - the best, 2 - intermediate, 3 - the worst)
______ 1st presentation
______ 2nd presentation
______ 3rd presentation

Rank_realistic Please rank the below presentations based on how realistic they were? (1 - the best, 2 - intermediate, 3 - the worst)
______ 1st presentation
______ 2nd presentation
______ 3rd presentation

Rank_overall Please rank the below presentations based on their overall effectiveness? (1 - the best, 2 - intermediate, 3 - the worst)

______ 1st presentation
______ 2nd presentation
______ 3rd presentation

Rank_format Please rank order the following presentation formats in terms of their general effectiveness, ignoring how well individual presenters (including today's) use that format. (1 - the best, 2 - intermediate, 3 - the worst)

______ Prezi
______ PowerPoint
______ Oral presentation

Rank_format_OE Considering your answer to the previous question, please explain why you ordered the presentation formats as you did.

Feedback Do you have any general feedback about the study?
