# Supplementary material for: Does a presentation’s medium affect its message? PowerPoint, Prezi, and oral presentations
Source: PLoS One. 2017 Jul 5;12(7):e0178774. doi: 10.1371/journal.pone.0178774 (PMC5497950; doi:10.1371/journal.pone.0178774)
Supplement: S3 File — (PDF) [file pone.0178774.s003.pdf]

Welcome!   Thank you for completing this brief questionnaire, which should take approximately 10 minutes to complete. Your thoughtfulness and honesty in answering these questions is extremely important to us.   Finally, it is important that you complete this survey in one sitting, and do not try to answer any of the questions by googling things in another browser window or tab.

ID Please tell us about yourself:

Contact email address:
Age (in years):
Current occupation:
Initials:

Education What is the highest level of education?

- ❍ No schooling completed (4)
- ❍ Nursery school to 8th grade (5)
- ❍ Some high school, no diploma (6)
- ❍ High school graduate, diploma or the equivalent (for example: GED) (7)
- ❍ Some college credit, no degree (8)
- ❍ Trade/technical/vocational training (9)
- ❍ Associate degree (10)
- ❍ Bachelor’s degree (11)
- ❍ Master’s degree (12)
- ❍ Professional degree (13)
- ❍ Doctorate degree (14)

Language Which of the below best describes you?

- ❍ I am a native English speaker
- ❍ I am not a native English speaker but fluent in English
- ❍ I am not a native English speaker and am not fluent in English

Gender What is your gender identity?

- ❍ Male (1)
- ❍ Female (2)
- ❍ Other (3)

A note on language: When we use the term "presentation", we mean a formal, planned, and oral presentation of any duration, including a public speech, an academic lecture, a webinar, a class presentation, a wedding toast, a sermon, a product demonstration, a business presentation, and so on. Examples of things we do NOT mean are: a theatrical performance, an impromptu toast at dinner, and any presentation with no audience. When we say PowerPoint presentations, we mean presentations that were made using Microsoft PowerPoint, not other software such as Apple's Keynote. Also, when we refer to "oral presentation", we mean a presentation that is only spoken and does not include any visual aids or the use of presentation software.

Experience How experienced are you at making the following types of presentations?

	Not at all Experienced (1)	Slightly Experienced (2)	Somewhat Experienced (3)	Very Experienced (4)	Extremely Experienced (5)
Oral Presentation	❍	❍	❍	❍	❍
PowerPoint	❍	❍	❍	❍	❍
Prezi	❍	❍	❍	❍	❍

Effective1 When you give a presentation, how effective are the following presentations for you?

	Not at all Effective (1)	Slightly Effective (2)	Somewhat Effective (3)	Very Effective (4)	Extremely Effective (5)
Oral Presentation	❍	❍	❍	❍	❍
PowerPoint	❍	❍	❍	❍	❍
Prezi	❍	❍	❍	❍	❍

Effective2 When somebody else gives a presentation, how effective are the following types of presentations for you?

	Not at all Effective (1)	Slightly Effective (2)	Somewhat Effective (3)	Very Effective (4)	Extremely Effective (5)
Oral Presentation	❍	❍	❍	❍	❍
PowerPoint	❍	❍	❍	❍	❍
Prezi	❍	❍	❍	❍	❍

ease How easy or difficult is it for you to make the following types of presentations?

	Not at all Difficult (1)	Slightly Difficult (3)	Somewhat Difficult (5)	Very Difficult (6)	Extremely Difficult (7)
Oral Presentation	❍	❍	❍	❍	❍
PowerPoint	❍	❍	❍	❍	❍
Prezi	❍	❍	❍	❍	❍

Freq_year In the last year, approximately how many of the following types of presentations did you make? (Please write "0" if you have not made any)

Oral Presentation:
PowerPoint:
Prezi:

Freq_life In your lifetime, approximately how many of the following types of presentations have you made? (Please write "0" if you have not made any)

Oral Presentation:
PowerPoint:
Prezi:

Year For approximately how many years have you been making the following types of presentations?

Oral Presentation:
PowerPoint:
Prezi:

Pros Proponents of the three types of presentations claim particular advantages for each type of presentation. In the table below, try to match the presentation type with its claimed advantages. You can only select one presentation type for each advantage.

	Oral Presentation	PowerPoint	Prezi
most easily allows you to create handouts and make notes to yourself	❍	❍	❍
most easily allows you to improvise during the presentation	❍	❍	❍
best shows relationships and connections between parts of your presentation	❍	❍	❍
most established, familiar, and reliable way to create visual aids	❍	❍	❍
best shows the big picture	❍	❍	❍
simplicity	❍	❍	❍
best promotes personal connection between you and your audience	❍	❍	❍
most fun, dynamic, and engaging for your audience	❍	❍	❍
most easily allows you to create tables and charts	❍	❍	❍

Cons Critics of the three types of presentations claim particular disadvantages of each type of presentation. In the table below, try to match presentation type with its claimed disadvantages. You can only select one presentation type for each disadvantage.

	Oral Presentation	PowerPoint	Prezi
makes it easy to overload your audience with information or distractions	❍	❍	❍
encourages overuse of bullet points and text	❍	❍	❍
expensive	❍	❍	❍
unsophisticated	❍	❍	❍
does not allow for visual communication or learning	❍	❍	❍
hard to learn	❍	❍	❍
provides no record of the presentation for later sharing or referencing	❍	❍	❍
encourages unoriginal, linear presentations	❍	❍	❍
can disorient your audience	❍	❍	❍

Functionality Please read the statements below and indicate whether or not Prezi and PowerPoint software has this functionality. You can select one, both, or neither software tool for each functionality.

	Prezi	PowerPoint
Add times to slides/frames	❑	❑
Use transformation tool to move, size and rotate content in your presentation	❑	❑
Create custom backgrounds	❑	❑
Use (+) button on the screen to zoom in	❑	❑
Record narration while presenting	❑	❑
Click "Present" button to start presenting	❑	❑
Use path points to set sequence of your presentation content	❑	❑
Insert videos from YouTube	❑	❑
Click on the objects to zoom around while editing your presentation	❑	❑
Import content from other presentations	❑	❑
Print handouts	❑	❑
Import presentations that were created using other presentation software	❑	❑
Double click to insert text	❑	❑
Stop presentation by pressing ESC	❑	❑

Next we will ask you to rate the quality of very short (10-20 second) samples of Prezi presentations. These videos have no sound or oral narration—this is on purpose so that you evaluate them based only on their visual aspects.

Prezi1 Please watch the four videos below, one at a time. When you are done watching them all, rank them from best to worst by clicking the button on the right side (1=best, 2 = good, 3 = bad, 4=worst). Feel free to watch the videos more than once if that helps you better evaluate them.

	1	2	3	4
Prezi1_video1https://s3.amazonaws.com/prz/Relation_1.mp4				
Prezi1_video2https://s3.amazonaws.com/prz/Relation_2.mp4				
Prezi1_video3https://s3.amazonaws.com/prz/Relation_3.mp4				

Prezi1_video4https://s3.amazonaws.com/prz/Relation_4.mp4				

Prezi2 Please watch the four videos below, one at a time. When you are done watching them all, rank them from best to worst by clicking the button on the right side (1=best, 2 = good, 3 = bad, 4=worst). Feel free to watch the videos more than once if that helps you better evaluate them.

	1	2	3	4
Prezi2_video1https://s3.amazonaws.com/prz/Zoom_1.mp4				
Prezi2_video2https://s3.amazonaws.com/prz/Zoom_2.mp4				
Prezi2_video3https://s3.amazonaws.com/prz/Zoom_3.mp4				
Prezi2_video4https://s3.amazonaws.com/prz/Zoom_4.mp4				

Prezi3 Please watch the four videos below, one at a time. When you are done watching them all, rank them from best to worst by clicking the button on the right side (1=best, 2 = good, 3 = bad, 4=worst). Feel free to watch the videos more than once if that helps you better evaluate them.

	1	2	3	4
Prezi3_video1https://s3.amazonaws.com/prz/metaphor_1.mp4				
Prezi3_video2https://s3.amazonaws.com/prz/metaphor_2.mp4				
Prezi3_video3https://s3.amazonaws.com/prz/metaphor_3.mp4				
Prezi3_video4https://s3.amazonaws.com/prz/metaphor_4.mp4				

Thank you again for completing the survey. You will receive an email from us within a few days.

ID Please fill in the form below.

ID (look at top right corner of the piece of paper you are given):

Initials:

In this activity, you will rate the quality of multiple presentations. A note on language: When we use the term "presentation", we mean a formal, planned, and oral presentation of any duration, including a public speech, an academic lecture, a webinar, a class presentation, a wedding toast, a sermon, a product demonstration, a business presentation, and so on. Examples of things we do NOT mean are: a theatrical performance, an impromptu toast at dinner, and any presentation with no audience. Also, when we refer to "oral presentation", we mean a presentation that is only spoken and does not include any visual aids or the use of presentation

software. Some presentations do not have narrative. This is on purpose. We want you to focus on the visual aspects on these presentations. Click next, when you are ready.

PPT1 Please watch the four videos below, one at a time. When you are done watching them all, rank them from best to worst by clicking the button on the right side (1=best, 2=good, 3=bad, 4=worst). Feel free to watch the videos more than once if that helps you better evaluate them.

	1	2	3	4
PPT1_video1https://s3.amazonaws.com/prz/Graphic_1.mp4				
PPT1_video2https://s3.amazonaws.com/prz/Graphic_2.mp4				
PPT1_video3https://s3.amazonaws.com/prz/Graphic_3.mp4				
PPT1_video4https://s3.amazonaws.com/prz/Graphic_4.mp4				

PPT2 Please watch the four videos below, one at a time. When you are done watching them all, rank them from best to worst by clicking the button on the right side (1=best, 2=good, 3=bad, 4=worst). Feel free to watch the videos more than once if that helps you better evaluate them.

	1	2	3	4
PPT2_video1https://s3.amazonaws.com/prz/charts_1.mp4				
PPT2_video2https://s3.amazonaws.com/prz/charts_2.mp4				
PPT2_video3https://s3.amazonaws.com/prz/charts_3.mp4				
PPT2_video4https://s3.amazonaws.com/prz/charts_4.mp4				

PPT3 Please watch the four videos below, one at a time. When you are done watching them all, rank them from best to worst by clicking the button on the right side (1=best, 2=good, 3=bad, 4=worst). Feel free to watch the videos more than once if that helps you better evaluate them.

	1	2	3	4
PPT3_video1<br>https://www.youtube.com/watch?v=T9NntA2Ny4A				
PPT3_video2<br>https://www.youtube.com/watch?v=tNL2nZ4nZ7c				
PPT3_video3<br>https://www.youtube.com/watch?v=atQSf4ON6Xs				
PPT3_video4<br>https://www.youtube.com/watch?v=liXffWyMhxk				

Speaking1 Please watch the four videos below, one at a time. When you are done watching them all, rank them from best to worst by clicking the button on the right side (1=best, 2=good, 3=bad, 4=worst). Feel free to watch the videos more than once if that helps you better evaluate them.

	1	2	3	4
Speaking1_video1<br>[video available by request]				
Speaking1_video2<br>[video available by request]				
Speaking1_video3<br>[video available by request]				
Speaking1_video4<br>[video available by request]				

Speaking2 Please watch the four videos below, one at a time. When you are done watching them all, rank them from best to worst by clicking the button on the right side (1=best, 2=good, 3=bad, 4=worst). Feel free to watch the videos more than once if that helps you better evaluate them.

	1	2	3	4
Speaking2_video1<br>[video available by request]				
Speaking2_video2<br>[video available by request]				
Speaking2_video3<br>[video available by request]				
Speaking2_video4<br>[video available by request]				

Speaking3 Please watch the four videos below, one at a time. When you are done watching them all, rank them from best to worst by clicking the button on the right side (1=best, 2=good, 3=bad, 4=worst). Feel free to watch the videos more than once if that helps you better evaluate them.

	1	2	3	4
Speaking3_video1<br>[video available by request]				
Speaking3_video2<br>[video available by request]				
Speaking3_video3<br>[video available by request]				
Speaking3_video4<br>[video available by request]				
