# Supplementary material for: Does a presentation’s medium affect its message? PowerPoint, Prezi, and oral presentations
Source: PLoS One. 2017 Jul 5;12(7):e0178774. doi: 10.1371/journal.pone.0178774 (PMC5497950; doi:10.1371/journal.pone.0178774)
Supplement: S4 File — (PDF) [file pone.0178774.s004.pdf]

ID Please fill in the form below.

ID (1)

Convince1 How convincing do you think your presentation will be to [Company X]'s board members?
- Not at all convincing (1)
- Slightly convincing (2)
- Somewhat convincing (3)
- Very convincing (4)
- Extremely convincing (5)

Convince2 How much do you personally agree with the argument you made in your presentation?
- Strongly disagree (1)
- Somewhat disagree (2)
- Slightly disagree (3)
- Neither agree nor disagree (4)
- Slightly agree (5)
- Somewhat agree (6)
- Strongly agree (7)

Convince3 What do you think [Company X] should do?
- Definitely rejecti-Mart's offer (1)
- Probably rejecti-Mart's offer (2)
- Possibly rejecti-Mart's offer (3)
- Possibly accepti-Mart's offer (4)
- Probably accepti-Mart's offer (5)
- Definitely accepti-Mart's offer (6)

Reason Why do you think [Company X] should accept or reject i-Mart's offer?

MetaCog1 You are now going to be quizzed on the content of the presentation you just made. Please rate how well you think you will perform on this quiz.
- Extremely poorly (1)
- Very Poorly (2)
- Poorly (4)
- Fair (5)
- Well (6)
- Very Well (7)
- Extremely Well (8)

MetaCog2 How much do you think you understand the situation with [Company X] and i-Mart?

- Not at all (1)
- Slightly (2)
- Somewhat (3)
- Mostly (4)
- Fully (5)

Relation1 How much do you think [Company X]'s online community is related its other aspects below?

	Not at all related (1)	Slightly related (2)	Somewhat related (3)	Very related (4)	Extremely related (5)
corporate culture	❍	❍	❍	❍	❍
product development	❍	❍	❍	❍	❍
financial success	❍	❍	❍	❍	❍
business model	❍	❍	❍	❍	❍
product marketing	❍	❍	❍	❍	❍

Relation2 How much do you think [Company X]'s corporate culture is related its other aspects below?

	Not at all related (1)	Slightly related (2)	Somewhat related (3)	Very related (4)	Extremely related (5)
product development	❍	❍	❍	❍	❍
financial success	❍	❍	❍	❍	❍
business model	❍	❍	❍	❍	❍
product marketing	❍	❍	❍	❍	❍

Relation3 How much do you think [Company X]'s product development is related its other aspects below?

	Not at all related (1)	Slightly related (2)	Somewhat related (3)	Very related (4)	Extremely related (5)
financial success	❍	❍	❍	❍	❍
business model	❍	❍	❍	❍	❍
product marketing	❍	❍	❍	❍	❍

Relation4 How much do you think [Company X]'s financial success is related its other aspects below?

	Not at all related (1)	Slightly related (2)	Somewhat related (3)	Very related (4)	Extremely related (5)
business model	❍	❍	❍	❍	❍
product marketing	❍	❍	❍	❍	❍

Relation5 How much do you think [Company X]'s business model is related its other aspects below?

	Not at all related (1)	Slightly related (2)	Somewhat related (3)	Very related (4)	Extremely related (5)
product marketing	❍	❍	❍	❍	❍

Relation6 How does [Company X]'s online community relate to its business model?

Relation7 What is the role of the Street Team program in [Company X]'s financial success?

Buss_Prces Below are the different steps of [Company X]' business process. Drag and drop the steps in order.
______ Design selection (1)
______ Design submission review (2)
______ Content management system (3)
______ Proofing and printing (4)
______ Distribution (5)

KeyElement According to [Company X], which of the following are key elements of its corporate culture (choose all apply)?
❑ Communication
❑ Fairness
❑ Transparency
❑ Fun
❑ Teamwork
❑ Hard work
❑ Deadlines authorized by the managers
❑ Employee autonomy

Community According to [Company X], which of the following is something its online community CANNOT do (choose all the apply)?

- ❑ Create designs for t-shirts
- ❑ Vote on designs
- ❑ Buy t-shirts
- ❑ Dislike others' designs
- ❑ Recommend t-shirts to other people online
- ❑ Earn credits for future [Company X] purchases
- ❑ Write on the [Company X] blog
- ❑ Impact [Company X] leadership's the decision making about the future of the company
- ❑ Communicate with [Company X] employees
- ❑ Fire [Company X] employees

Int_Motiv We would like you to reflect back to your experience of creating your presentation. For each of the following statements, please indicate how true it is for you.

	1 (1)	2 (2)	3 (3)	4 (4)	5 (5)	6 (6)	7 (7)
I enjoyed doing this activity very much	❍	❍	❍	❍	❍	❍	❍
This activity was fun to do.	❍	❍	❍	❍	❍	❍	❍
I thought this was a boring activity.	❍	❍	❍	❍	❍	❍	❍
This activity did not hold my attention at all.	❍	❍	❍	❍	❍	❍	❍
I would describe this activity as very interesting.	❍	❍	❍	❍	❍	❍	❍
I thought this activity was quite enjoyable.	❍	❍	❍	❍	❍	❍	❍
While I was doing this activity, I was thinking about how much I enjoyed it.	❍	❍	❍	❍	❍	❍	❍
I think I am pretty good at this activity.	❍	❍	❍	❍	❍	❍	❍
I think I did pretty well at this activity, compared to other presenters.	❍	❍	❍	❍	❍	❍	❍
After working at this activity	❍	❍	❍	❍	❍	❍	❍

for awhile, I felt pretty competent.							
I am satisfied with my performance at this task.	❍	❍	❍	❍	❍	❍	❍
I was pretty skilled at this activity.	❍	❍	❍	❍	❍	❍	❍
This was an activity that I couldn't do very well.	❍	❍	❍	❍	❍	❍	❍
I put a lot of effort into this.	❍	❍	❍	❍	❍	❍	❍
I didn't try very hard to do well at this activity. (R)	❍	❍	❍	❍	❍	❍	❍
I tried very hard on this activity.	❍	❍	❍	❍	❍	❍	❍
It was important to me to do well at this task.	❍	❍	❍	❍	❍	❍	❍
I didn't put much energy into this. (R)	❍	❍	❍	❍	❍	❍	❍

Interest How interested are you in making the presentations in the future using the tool or the format you used today?

- ❍ Extremely uninterested (1)
- ❍ Somewhat uninterested (2)
- ❍ Slightly uninterested (3)
- ❍ Neutral (4)
- ❍ Slightly interested (5)
- ❍ Somewhat interested (6)
- ❍ Extremely interested (7)

Recommend How likely are you to recommend the presentation tool or presentation format you used to others to make professional presentations?

- ❍ Extremely unlikely (1)
- ❍ Somewhat unlikely (2)
- ❍ Slightly unlikely (3)
- ❍ Neutral (4)
- ❍ Slightly likely (5)
- ❍ Somewhat likely (6)
- ❍ Extremely likely (7)
