# Supplementary material for: Does a presentation’s medium affect its message? PowerPoint, Prezi, and oral presentations
Source: PLoS One. 2017 Jul 5;12(7):e0178774. doi: 10.1371/journal.pone.0178774 (PMC5497950; doi:10.1371/journal.pone.0178774)
Supplement: S5 File — (PDF) [file pone.0178774.s005.pdf]

Hello Thank you for participating in this study!  Please tell us a little bit about yourself.

MTurk ID Please enter your MTurk ID below:

Gender What is your gender?
- Female (1)
- Male (2)

Age How old are you (in years)? Please enter only a number.
If How old are you?   Is Less Than 18, Then Skip To End of Survey

Language Are you a native English speaker?
- Yes (1)
- No (2)

If no Is Selected, Then Skip To End of Survey

Education What is the highest level of education you completed?
- Did not complete high school (1)
- High school (or equivalent) (2)
- Some college (or associate degree) (3)
- Bachelor's degree (4)
- Master's degree (5)
- Doctoral or professional degree (6)

Profession If you are employed, what is your profession? (if you are not employed, please skip this question)

Buss-experience How experienced are you with concepts and practices from the business world, such as strategy, innovation, product development, sales, and marketing?
- Not at all experienced (1)
- Slightly experienced (2)
- Somewhat experienced (3)
- Very experienced (4)
- Extremely experienced (5)

Student? Are you currently a student in a degree program?
- Yes. I am pursuing a degree in: (1) ____________________
- No (0)

[*Present background material*]

Familiarity How familiar are you with the company [Company X]?
- ❍ Not at all familiar (1)
- ❍ Slightly familiar (2)
- ❍ Somewhat familiar (3)
- ❍ Very familiar (4)
- ❍ Extremely familiar (5)

Trap1 What product does [Company X] company sell?
- ❍ Cars (0)
- ❍ T-shirts (1)
- ❍ Backpacks (0)
- ❍ Computers (0)
- ❍ Socks (0)

If T-shirts Is Not Selected, Then Skip To End of Survey

Affective1

| | Not at all (1) | A little (2) | Somewhat (3) | Very much (4) | Completely (5) |
| --- | --- | --- | --- | --- | --- |
| How engaging did you find this background material? | ❍ | ❍ | ❍ | ❍ | ❍ |
| How much did you enjoy this background material? | ❍ | ❍ | ❍ | ❍ | ❍ |
| How much did you pay attention to this background material? | ❍ | ❍ | ❍ | ❍ | ❍ |
| How difficult was it for you to understand this background material? | ❍ | ❍ | ❍ | ❍ | ❍ |

[*G4 is the control group*]

[Participants are randomly shown one of the three videos]

Info-g4vd1 Before you decide what to do about i-Mart's offer to [Company X], we would like you to watch an unrelated presentation and briefly answer some questions about it.

G4-video_1 https://s3.amazonaws.com/prz2/G4_V1.mp4

Info-g4vd2 Before you decide what to do about i-Mart's offer to [Company X], we would like you to watch an unrelated presentation and briefly answer some questions about it.

G4_video_2 https://s3.amazonaws.com/prz2/G4_V2.mp4

Info-g4vd3 Before you decide what to do about i-Mart's offer to [Company X], we would like you to watch an unrelated presentation and briefly answer some questions about it.

G4_video_3 https://s3.amazonaws.com/prz2/G4_V3.mp4

G4-affect

| | Not at all (1) | A little (2) | Somewhat (3) | Very much (4) | Completely (5) |
|---|---|---|---|---|---|
| How engaging did you find this video? | ❍ | ❍ | ❍ | ❍ | ❍ |
| How much did you enjoy this video? | ❍ | ❍ | ❍ | ❍ | ❍ |
| How much did you pay attention to this video? | ❍ | ❍ | ❍ | ❍ | ❍ |
| How difficult was it for you to understand this video? | ❍ | ❍ | ❍ | ❍ | ❍ |

G4-overall Overall, how ______ was this presentation?

| | Not at all (1) | Slightly (2) | Somewhat (3) | Very (4) | Extremely (5) |
|---|---|---|---|---|---|
| Organized | ❍ | ❍ | ❍ | ❍ | ❍ |
| Engaging | ❍ | ❍ | ❍ | ❍ | ❍ |
| Persuasive | ❍ | ❍ | ❍ | ❍ | ❍ |
| Effective | ❍ | ❍ | ❍ | ❍ | ❍ |

G4-present Overall, how ______ was this presenter?

| | Not at all (1) | Slightly (2) | Somewhat (3) | Very (4) | Extremely (5) |
|---|---|---|---|---|---|
| Organized | ❍ | ❍ | ❍ | ❍ | ❍ |
| Engaging | ❍ | ❍ | ❍ | ❍ | ❍ |
| Persuasive | ❍ | ❍ | ❍ | ❍ | ❍ |
| Effective | ❍ | ❍ | ❍ | ❍ | ❍ |
| Confident | ❍ | ❍ | ❍ | ❍ | ❍ |
| Enthusiastic | ❍ | ❍ | ❍ | ❍ | ❍ |
| Knowledgeable | ❍ | ❍ | ❍ | ❍ | ❍ |
| Professional | ❍ | ❍ | ❍ | ❍ | ❍ |
| Nervous | ❍ | ❍ | ❍ | ❍ | ❍ |
| Boring | ❍ | ❍ | ❍ | ❍ | ❍ |

G4-media Overall, how much did this presentation material have the following?

| | Not enough (1) | About right (2) | Too much (3) |
|---|---|---|---|
| Text | ❍ | ❍ | ❍ |
| Graphs | ❍ | ❍ | ❍ |
| Images | ❍ | ❍ | ❍ |
| Animations (e.g., zooming) | ❍ | ❍ | ❍ |

G4-summary Please summarize the presentation. What were the main points? (minimum acceptable length for the answer - 50 characters)

G4-comment Do you have any comments about the presenter? How do you think s/he can improve the presentation?(minimum acceptable length for the answer - 50 characters)

MetaCog1 How much do you think you understand the situation with [Company X] and i-Mart?
- Not at all (1)
- Slightly (2)
- Somewhat (3)
- Mostly (4)
- Fully (5)

MetaCog1 You are now going to be quizzed on the content you just studied. Please rate how well you think you will perform on this quiz.
- Extremely Poorly (1)
- Very Poorly (2)
- Somewhat Poorly (3)
- Fair (4)
- Somewhat Well (5)
- Very Well (6)
- Extremely Well (7)

Decision1 What do you think [Company X] should do?
- Definitely rejecti-Mart's offer (1)
- Probably rejecti-Mart's offer (2)
- Possibly rejecti-Mart's offer (3)
- Possibly accepti-Mart's offer (4)
- Probably accepti-Mart's offer (5)
- Definitely accepti-Mart's offer (6)

Reason1 Why do you think [Company X] should accept or reject i-Mart's offer? (minimum acceptable length for the answer - 100 characters)

Pro1 What are the pros of accepting i-Mart's offer? (minimum acceptable length for the answer - 50 characters)

Con1 What are the cons of accepting i-Mart's offer? (minimum acceptable length for the answer - 50 characters)

Fact-# According to the background materials and the presentation, approximately how many members does [Company X] have?
- 500,000 (1)
- 150,000 (0)
- 25,000 (0)
- 3,500 (0)
- 1.5 million (0)

Fact-state [Company X]'s customer base is concentrated in:
- The South (0)
- The East Coast (0)
- The West Coast (0)
- Both the East and West Coast (1)
- The Midwest (0)

Fact-proft Between 2000 and 2007, [Company X]'s revenue:
- Decreased dramatically (0)
- Decreased somewhat (0)
- Stayed the same (0)
- Increased somewhat (0)
- Increased dramatically (1)

Fact-chall According to the background materials, what is the biggest challenge [Company X] is facing?
- Marketing (0)
- Maintaining transparency (0)
- Community expansion (0)
- Logistics (1)
- Employee satisfaction (0)

KeyAspect1 How important are the below aspects of [Company X]'s corporate culture?

| | Not at all important (1) | Slightly important (2) | Somewhat important (3) | Very important (4) | Extremely important (5) |
|---|---|---|---|---|---|
| Openness and communication | ❍ | ❍ | ❍ | ❍ | ❍ |
| Trust and accountability | ❍ | ❍ | ❍ | ❍ | ❍ |
| Fun and hard work | ❍ | ❍ | ❍ | ❍ | ❍ |
| Customer service | ❍ | ❍ | ❍ | ❍ | ❍ |
| Health and wellbeing | ❍ | ❍ | ❍ | ❍ | ❍ |
| Environmental sustainability | ❍ | ❍ | ❍ | ❍ | ❍ |
| Diversity and inclusion | ❍ | ❍ | ❍ | ❍ | ❍ |

Trap2 Are you a human? Please answer the following math question: 84 plus 27 =

Reason1 For each of the below items, please indicate whether you think it is a reason for [Company X] to accept i-Mart's offer, reject i-Mart's offer, or neither.

| | reason to accept offer | reason to reject offer | neither |
|---|---|---|---|
| It's a way to advertise, to bring people to the site | ❍ | ❍ | ❍ |
| Migrating to a new medium is a natural development | ❍ | ❍ | ❍ |
| It's a way to outsource distribution to a retailer | ❍ | ❍ | ❍ |
| It's a grown up move, a way for [Company X] to mature as a company | ❍ | ❍ | ❍ |
| [Company X] community might become less exclusive | ❍ | ❍ | ❍ |
| Existing community members might think that [Company X] is a "sellout" | ❍ | ❍ | ❍ |
| Designs and community might become less creative | ❍ | ❍ | ❍ |
| [Company X] might become less democratic | ❍ | ❍ | ❍ |
| i-Mart might bias choices of t-shirts | ❍ | ❍ | ❍ |
| Company culture is not suited to deal with i-Mart | ❍ | ❍ | ❍ |
| Employees don't want to work for company that is just a supplier to i-Mart | ❍ | ❍ | ❍ |
| Logistics: [Company X] won't be able to handle the increased | ❍ | ❍ | ❍ |

| | | | |
|---|---|---|---|
| production demand | | | |
| Company might be flooded with inferior design submissions | ❍ | ❍ | ❍ |
| [Company X] will sell more t-shirts and increase profits | ❍ | ❍ | ❍ |
| The community is worth more than selling more t-shirts | ❍ | ❍ | ❍ |
| Designers will have a broader audience for their work | ❍ | ❍ | ❍ |

Relate1.1 How much do you think [Company X]'s online community is related its other aspects below?

| | Not at all related (1) | Slightly related (2) | Somewhat related (3) | Very related (4) | Extremely related (5) |
|---|---|---|---|---|---|
| corporate culture | ❍ | ❍ | ❍ | ❍ | ❍ |
| product development | ❍ | ❍ | ❍ | ❍ | ❍ |
| financial success | ❍ | ❍ | ❍ | ❍ | ❍ |
| business model | ❍ | ❍ | ❍ | ❍ | ❍ |
| product marketing | ❍ | ❍ | ❍ | ❍ | ❍ |

Relate1.2 How much do you think [Company X]'s corporate culture is related its other aspects below?

| | Not at all related (1) | Slightly related (2) | Somewhat related (3) | Very related (4) | Extremely related (5) |
|---|---|---|---|---|---|
| product development | ❍ | ❍ | ❍ | ❍ | ❍ |
| financial success | ❍ | ❍ | ❍ | ❍ | ❍ |
| business model | ❍ | ❍ | ❍ | ❍ | ❍ |
| product marketing | ❍ | ❍ | ❍ | ❍ | ❍ |

Relate1.3 How much do you think [Company X]'s product development is related its other aspects below?

| | Not at all related (1) | Slightly related (2) | Somewhat related (3) | Very related (4) | Extremely related (5) |
|---|---|---|---|---|---|
| financial success | ❍ | ❍ | ❍ | ❍ | ❍ |
| business model | ❍ | ❍ | ❍ | ❍ | ❍ |
| product marketing | ❍ | ❍ | ❍ | ❍ | ❍ |

Relate1.4 How much do you think [Company X]'s financial success is related its other aspects below?

| | Not at all related (1) | Slightly related (2) | Somewhat related (3) | Very related (4) | Extremely related (5) |
|---|---|---|---|---|---|
| business model | ❍ | ❍ | ❍ | ❍ | ❍ |
| product marketing | ❍ | ❍ | ❍ | ❍ | ❍ |

Relate1.5 How much do you think [Company X]'s business model is related its product marketing?

| | Not at all related (1) | Slightly related (2) | Somewhat related (3) | Very related (4) | Extremely related (5) |
|---|---|---|---|---|---|
| product marketing | ❍ | ❍ | ❍ | ❍ | ❍ |

Relate1.6 How does [Company X]' online community relate to its business model?(minimum acceptable length for the answer - 50 characters)

eval-all Overall, how ______ was this presentation?

| | Not at all (1) | Slightly (2) | Somewhat (3) | Very (4) | Extremely (5) |
|---|---|---|---|---|---|
| Organized | ❍ | ❍ | ❍ | ❍ | ❍ |
| Engaging | ❍ | ❍ | ❍ | ❍ | ❍ |
| Realistic | ❍ | ❍ | ❍ | ❍ | ❍ |
| Persuasive | ❍ | ❍ | ❍ | ❍ | ❍ |
| Effective | ❍ | ❍ | ❍ | ❍ | ❍ |

Answer If Group Is Equal to 1 Or Group Is Equal to 2

eval-visual Overall, how ______ was the visual component of this presentation (that appeared on the left half of the screen)?

| | Not at all (1) | Slightly (2) | Somewhat (3) | Very (4) | Extremely (5) |
|---|---|---|---|---|---|
| Organized | ❍ | ❍ | ❍ | ❍ | ❍ |
| Engaging | ❍ | ❍ | ❍ | ❍ | ❍ |
| Persuasive | ❍ | ❍ | ❍ | ❍ | ❍ |
| Effective | ❍ | ❍ | ❍ | ❍ | ❍ |
| Dynamic | ❍ | ❍ | ❍ | ❍ | ❍ |
| Visually compelling | ❍ | ❍ | ❍ | ❍ | ❍ |
| Distracting | ❍ | ❍ | ❍ | ❍ | ❍ |
| Informative | ❍ | ❍ | ❍ | ❍ | ❍ |
| Distinctive | ❍ | ❍ | ❍ | ❍ | ❍ |
| Boring | ❍ | ❍ | ❍ | ❍ | ❍ |

eval-person Overall, how ______ was this presenter (who appeared on the right half of the screen)?

| | Not at all (1) | Slightly (2) | Somewhat (3) | Very (4) | Extremely (5) |
|---|---|---|---|---|---|
| Organized | ❍ | ❍ | ❍ | ❍ | ❍ |
| Engaging | ❍ | ❍ | ❍ | ❍ | ❍ |
| Persuasive | ❍ | ❍ | ❍ | ❍ | ❍ |
| Effective | ❍ | ❍ | ❍ | ❍ | ❍ |
| Confident | ❍ | ❍ | ❍ | ❍ | ❍ |
| Enthusiastic | ❍ | ❍ | ❍ | ❍ | ❍ |
| Knowledgeable | ❍ | ❍ | ❍ | ❍ | ❍ |
| Professional | ❍ | ❍ | ❍ | ❍ | ❍ |
| Nervous | ❍ | ❍ | ❍ | ❍ | ❍ |
| Boring | ❍ | ❍ | ❍ | ❍ | ❍ |

Answer If  Group Is Equal to  1 Or  Group Is Equal to  2
eval-media Overall, how much did this presentation material have the following?

| | Not enough (-1) | About right (0) | Too much (1) |
|---|---|---|---|
| Text | ❍ | ❍ | ❍ |
| Graphs | ❍ | ❍ | ❍ |
| Images | ❍ | ❍ | ❍ |
| Animations (e.g., zooming) | ❍ | ❍ | ❍ |

Summarize Please summarize the presentation. What were the main selling points?(minimum acceptable length for the answer - 50 characters)

OE_person Do you have any comments about the presenter? How do you think s/he can improve the presentation?(minimum acceptable length for the answer - 50 characters)

Answer If  Group Is Equal to  1 Or  Group Is Equal to  2
OE_visuals Do you have any comments about the visual part of this presentation (that appeared on the left half of the video)? How do you think it can be improved? (minimum acceptable length for the answer - 50 characters)

Vid-Quality Please rate the quality of the presentation's audio and video.

| | Very Bad (1) | Bad (2) | Okay (3) | Good (4) | Very Good (5) |
|---|---|---|---|---|---|
| Audio | ❍ | ❍ | ❍ | ❍ | ❍ |
| Video | ❍ | ❍ | ❍ | ❍ | ❍ |

Answer If Please rate the quality of the presentation's audio and video.  - Very Bad Is Selected Or Please rate the quality of the presentation's audio and video.  - Bad Is Selected
Vid-Qual2 What problems were there with the audio or video?

Transition Awesome! In the next part of the study, we would like to know your experiences with different types of presentations.

Note A note on language: When we use the term "presentation", we mean a formal, planned, and oral presentation of any duration, including a public speech, an academic lecture, a webinar, a class presentation, a wedding toast, a sermon, a product demonstration, a business presentation, and so on. Examples of things we do NOT mean are: a theatrical performance, an impromptu toast at dinner, and any presentation with no audience .When we say PowerPoint presentations, we mean presentations that

were made using Microsoft PowerPoint, not other software such as Apple's Keynote. When we say Prezi presentations, we mean presentations that were made using Prezi presentation software. Also, when we refer to "oral presentation", we mean a presentation that is only spoken and does not include any visual aids or the use of presentation software.

Exp-M_1 How experienced are you at making the following types of presentations?

| | Not at all Experienced (1) | Slightly Experienced (2) | Somewhat Experienced (3) | Very Experienced (4) | Extremely Experienced (5) |
|---|---|---|---|---|---|
| Oral Presentation | ❍ | ❍ | ❍ | ❍ | ❍ |
| PowerPoint | ❍ | ❍ | ❍ | ❍ | ❍ |
| Prezi | ❍ | ❍ | ❍ | ❍ | ❍ |

Exp-M_2 Generally speaking, how effective are the following types of presentations in your opinion?

| | Extremely Ineffective (1) | Somewhat Ineffective (2) | Slightly Ineffective (3) | Neither Effective nor Ineffective (4) | Slightly Effective (5) | Somewhat Effective (6) | Extremely Effective (7) | N Appl (9 |
|---|---|---|---|---|---|---|---|---|
| Oral Presentation | ❍ | ❍ | ❍ | ❍ | ❍ | ❍ | ❍ | ❍ |
| PowerPoint | ❍ | ❍ | ❍ | ❍ | ❍ | ❍ | ❍ | ❍ |
| Prezi | ❍ | ❍ | ❍ | ❍ | ❍ | ❍ | ❍ | ❍ |

Exp-M_3 How effective are you at making the following types of presentations?

| | Extremely Ineffective (1) | Somewhat Ineffective (2) | Slightly Ineffective (3) | Neither Effective nor Ineffective (4) | Slightly Effective (5) | Somewhat Effective (6) | Extremely Effective (7) | N Appl (9 |
|---|---|---|---|---|---|---|---|---|
| Oral Presentation | ❍ | ❍ | ❍ | ❍ | ❍ | ❍ | ❍ | ❍ |
| PowerPoint | ❍ | ❍ | ❍ | ❍ | ❍ | ❍ | ❍ | ❍ |
| Prezi | ❍ | ❍ | ❍ | ❍ | ❍ | ❍ | ❍ | ❍ |

Exp_M_4 How easy or difficult is it for you to make the following types of presentations?

| | Extremely Difficult (1) | Somewhat Difficult (2) | Slightly Difficult (3) | Neither Easy nor Difficult (4) | Slightly Easy (5) | Somewhat Easy (6) | Extremely Easy (7) | Not Applicable (99) |
|---|---|---|---|---|---|---|---|---|
| Oral Presentation | ❍ | ❍ | ❍ | ❍ | ❍ | ❍ | ❍ | ❍ |
| PowerPoint | ❍ | ❍ | ❍ | ❍ | ❍ | ❍ | ❍ | ❍ |
| Prezi | ❍ | ❍ | ❍ | ❍ | ❍ | ❍ | ❍ | ❍ |

Exp_M_5 In the last year, approximately how many of the following types of presentations did you make? (Please write "0" if you have not made any)

Oral Presentation
PowerPoint
Prezi

Exp_M_6 In your lifetime, approximately how many of the following types of presentations have you made? (Please write "0" if you have not made any)

Oral Presentation
PowerPoint
Prezi

Exp_M_7 For approximately how many years have you been making the following types of presentations? (Please write "0" if you have not made any)

Oral Presentation
PowerPoint
Prezi

Effect_W_1 When you give a presentation, how effective are the following types of presentations for you?

| | Not at all Effective (1) | Slightly Effective (2) | Somewhat Effective (3) | Very Effective (4) | Extremely Effective (5) | Not Applicable (99) |
|---|---|---|---|---|---|---|
| Oral Presentation | ❍ | ❍ | ❍ | ❍ | ❍ | ❍ |
| PowerPoint | ❍ | ❍ | ❍ | ❍ | ❍ | ❍ |
| Prezi | ❍ | ❍ | ❍ | ❍ | ❍ | ❍ |

Effect_W_2 When somebody else gives a presentation, how effective are the following types of presentations for you?

| | Not at all Effective (1) | Slightly Effective (2) | Somewhat Effective (3) | Very Effective (4) | Extremely Effective (5) | Not Applicable (99) |
|---|---|---|---|---|---|---|
| Oral Presentation | ❍ | ❍ | ❍ | ❍ | ❍ | ❍ |
| PowerPoint | ❍ | ❍ | ❍ | ❍ | ❍ | ❍ |
| Prezi | ❍ | ❍ | ❍ | ❍ | ❍ | ❍ |

Effect_W_3 When somebody else gives a presentation, how enjoyable are the following types of presentations for you?

| | Not at all Enjoyable (1) | Slightly Enjoyable (2) | Somewhat Enjoyable (3) | Very Enjoyable (4) | Extremely Enjoyable (5) | Not Applicable (99) |
|---|---|---|---|---|---|---|
| Oral Presentation | ❍ | ❍ | ❍ | ❍ | ❍ | ❍ |
| PowerPoint | ❍ | ❍ | ❍ | ❍ | ❍ | ❍ |
| Prezi | ❍ | ❍ | ❍ | ❍ | ❍ | ❍ |

Freq_1 In the last year, approximately how many of the following types of presentations did watch? (Please write "0" if you have not watched any)

Oral Presentation:
PowerPoint:
Prezi:

Freq_2 In your lifetime, approximately how many of the following types of presentations did you watch? (Please write "0" if you have not watched any)

Oral Presentation:
PowerPoint:
Prezi:

Years For approximately how many years have you been watching the following types of presentations? Please write "0" if you have not watched any)

Oral Presentation:
PowerPoint:
Prezi:

Advantage Proponents of the three types of presentations claim particular advantages for each type of presentation. In the table below, try to match the presentation type with its claimed advantages. You can only select one presentation type for each advantage.

| | Oral Presentation | PowerPoint | Prezi |
|---|---|---|---|
| most easily allows you to create handouts and make notes to yourself | ❍ | ❍ | ❍ |
| most easily allows you to improvise during the presentation | ❍ | ❍ | ❍ |
| best shows relationships and connections between parts of your presentation | ❍ | ❍ | ❍ |
| most established, familiar, and reliable way to create visual aids | ❍ | ❍ | ❍ |
| best shows the big picture | ❍ | ❍ | ❍ |
| simplicity | ❍ | ❍ | ❍ |
| best promotes personal connection between you and your audience | ❍ | ❍ | ❍ |
| most fun, dynamic, and engaging for your audience | ❍ | ❍ | ❍ |
| most easily allows you to create tables and charts | ❍ | ❍ | ❍ |

Disadvantage Critics of the three types of presentations claim particular disadvantages of each type of presentation. In the table below, try to match presentation type with its claimed disadvantages. You can only select one presentation type for each disadvantage.

| | Oral Presentation | PowerPoint | Prezi |
|---|---|---|---|
| makes it easy to overload your audience with information or distractions | ❍ | ❍ | ❍ |
| encourages overuse of bullet points and text | ❍ | ❍ | ❍ |
| expensive | ❍ | ❍ | ❍ |
| unsophisticated | ❍ | ❍ | ❍ |
| does not allow for visual communication or learning | ❍ | ❍ | ❍ |
| hard to learn | ❍ | ❍ | ❍ |
| provides no record of the presentation for later sharing or referencing | ❍ | ❍ | ❍ |
| encourages unoriginal, linear presentations | ❍ | ❍ | ❍ |
| can disorient your audience | ❍ | ❍ | ❍ |

Functionality Please read the statements below and indicate whether or not Prezi and PowerPoint software has this functionality. You can select one or both software tool for each functionality.

| | Prezi | PowerPoint |
|---|---|---|
| Add times to slides/frames | ❑ | ❑ |
| Use transformation tool to move, size and rotate content in your presentation | ❑ | ❑ |
| Create custom backgrounds | ❑ | ❑ |
| Use (+) button on the screen to zoom in | ❑ | ❑ |
| Record narration while presenting | ❑ | ❑ |
| Click "Present" button to start presenting | ❑ | ❑ |
| Use path points to set sequence of your presentation content | ❑ | ❑ |
| Insert videos from YouTube | ❑ | ❑ |
| Click on the objects to zoom around while editing your presentation | ❑ | ❑ |
| Import content from other presentations | ❑ | ❑ |
| Print handouts | ❑ | ❑ |
| Import presentations that were created using other presentation software | ❑ | ❑ |
| Double click to insert text | ❑ | ❑ |
| Stop presentation by pressing ESC | ❑ | ❑ |

Next, you will rate the quality of short presentations. Some presentations do not have narrative. This is on purpose. We want you to focus on the visual aspects on these presentations. Click next, when you are ready.

PPT1 Please watch the four videos below, one at a time. When you are done watching them all, rank them from best to worst by clicking the button on the right side (1=best, 2 = good, 3 = bad, 4=worst). Feel free to watch the videos more than once if that helps you better evaluate them.

| | 1 | 2 | 3 | 4 |
|---|---|---|---|---|
| PPT1_video1https://s3.amazonaws.com/prz/Graphic_1.mp4 | | | | |
| PPT1_video2https://s3.amazonaws.com/prz/Graphic_2.mp4 | | | | |
| PPT1_video3https://s3.amazonaws.com/prz/Graphic_3.mp4 | | | | |
| PPT1_video4https://s3.amazonaws.com/prz/Graphic_4.mp4 | | | | |

Note *The submit button is disabled for the time that will take you to view the videos.*

Speaking1 Please watch the four videos below, one at a time. When you are done watching them all, rank them from best to worst by clicking the button on the right side (1=best, 2=good, 3=bad, 4=worst). Feel free to watch the videos more than once if that helps you better evaluate them.

| | 1 | 2 | 3 | 4 |
|---|---|---|---|---|
| Speaking1_video1<br>[video available by request] | | | | |
| Speaking1_video2<br>[video available by request] | | | | |
| Speaking1_video3<br>[video available by request] | | | | |
| Speaking1_video4<br>[video available by request] | | | | |

Note *The submit button is disabled for the time that will take you to view the videos.*

Prezi1 Please watch the four videos below, one at a time. When you are done watching them all, rank them from best to worst by clicking the button on the right side (1=best, 2 = good, 3 = bad, 4=worst). Feel free to watch the videos more than once if that helps you better evaluate them.

| | 1 | 2 | 3 | 4 |
|---|---|---|---|---|
| Prezi1_video1https://s3.amazonaws.com/prz/Relation_1.mp4 | | | | |
| Prezi1_video2https://s3.amazonaws.com/prz/Relation_2.mp4 | | | | |
| Prezi1_video3https://s3.amazonaws.com/prz/Relation_3.mp4 | | | | |
| Prezi1_video4https://s3.amazonaws.com/prz/Relation_4.mp4 | | | | |

Note *The submit button is disabled for the time that will take you to view the videos.*

PPT2 Please watch the four videos below, one at a time. When you are done watching them all, rank them from best to worst by clicking the button on the right side (1=best, 2 = good, 3 = bad, 4=worst). Feel free to watch the videos more than once if that helps you better evaluate them.

| | 1 | 2 | 3 | 4 |
|---|---|---|---|---|
| PPT2_video1https://s3.amazonaws.com/prz/charts_1.mp4 | | | | |
| PPT2_video2https://s3.amazonaws.com/prz/charts_2.mp4 | | | | |
| PPT2_video3https://s3.amazonaws.com/prz/charts_3.mp4 | | | | |
| PPT2_video4https://s3.amazonaws.com/prz/charts_4.mp4 | | | | |

Note *The submit button is disabled for the time that will take you to view the videos.*

Speaking2 Please watch the four videos below, one at a time. When you are done watching them all, rank them from best to worst by clicking the button on the right side (1=best, 2=good, 3=bad, 4=worst). Feel free to watch the videos more than once if that helps you better evaluate them.

| | 1 | 2 | 3 | 4 |
|---|---|---|---|---|
| Speaking2_video1 [video available by request] | | | | |
| Speaking2_video2 [video available by request] | | | | |
| Speaking2_video3 [video available by request] | | | | |
| Speaking2_video4 [video available by request] | | | | |

Note *The submit button is disabled for the time that will take you to view the videos.*

Prezi2 Please watch the four videos below, one at a time. When you are done watching them all, rank them from best to worst by clicking the button on the right side (1=best, 2 = good, 3 = bad, 4=worst). Feel free to watch the videos more than once if that helps you better evaluate them.

| | 1 | 2 | 3 | 4 |
|---|---|---|---|---|
| Prezi2_video1https://s3.amazonaws.com/prz/Zoom_1.mp4 | | | | |
| Prezi2_video2https://s3.amazonaws.com/prz/Zoom_2.mp4 | | | | |
| Prezi2_video3https://s3.amazonaws.com/prz/Zoom_3.mp4 | | | | |
| Prezi2_video4https://s3.amazonaws.com/prz/Zoom_4.mp4 | | | | |

Note *The submit button is disabled for the time that will take you to view the videos.*

PPT3 Please watch the four videos below, one at a time. When you are done watching them all, rank them from best to worst by clicking the button on the right side (1=best, 2=good, 3=bad, 4=worst). Feel free to watch the videos more than once if that helps you better evaluate them.

| | 1 | 2 | 3 | 4 |
|---|---|---|---|---|
| PPT3_video1 https://www.youtube.com/watch?v=T9NntA2Ny4A | | | | |
| PPT3_video2 https://www.youtube.com/watch?v=tNL2nZ4nZ7c | | | | |
| PPT3_video3 https://www.youtube.com/watch?v=atQSf4ON6Xs | | | | |
| PPT3_video4 https://www.youtube.com/watch?v=liXffWyMhxk | | | | |

Note *The submit button is disabled for the time that will take you to view the videos.*

Speaking3 Please watch the four videos below, one at a time. When you are done watching them all, rank them from best to worst by clicking the button on the right side (1=best, 2=good, 3=bad, 4=worst). Feel free to watch the videos more than once if that helps you better evaluate them.

| | 1 | 2 | 3 | 4 |
|---|---|---|---|---|
| Speaking3_video1 [video available by request] | | | | |
| Speaking3_video2 [video available by request] | | | | |
| Speaking3_video3 [video available by request] | | | | |
| Speaking3_video4 [video available by request] | | | | |

Note *The submit button is disabled for the time that will take you to view the videos.*

Prezi3 Please watch the four videos below, one at a time. When you are done watching them all, rank them from best to worst by clicking the button on the right side (1=best, 2 = good, 3 = bad, 4=worst). Feel free to watch the videos more than once if that helps you better evaluate them.

| | 1 | 2 | 3 | 4 |
|---|---|---|---|---|
| Prezi3_video1https://s3.amazonaws.com/prz/metaphor_1.mp4 | | | | |
| Prezi3_video2https://s3.amazonaws.com/prz/metaphor_2.mp4 | | | | |
| Prezi3_video3https://s3.amazonaws.com/prz/metaphor_3.mp4 | | | | |
| Prezi3_video4https://s3.amazonaws.com/prz/metaphor_4.mp4 | | | | |

Note *The submit button is disabled for the time that will take you to view the videos.*
